# The balancing act between high electronic and low ionic transport influenced by perovskite grain boundaries†

**DOI:** 10.1039/d3ta04458k

**Published:** 2024-03-21

**Authors:** Nadja Glück, Nathan S. Hill, Marcin Giza, Eline Hutter, Irene Grill, Johannes Schlipf, Udo Bach, Peter Müller-Buschbaum, Achim Hartschuh, Thomas Bein, Tom Savenije, Pablo Docampo

**Affiliations:** a Department of Chemistry and Center for NanoScience (CeNS), University of Munich (LMU) Butenandtstr. 5-13 81377 München Germany; b Department of Chemical Engineering, Monash University Clayton Victoria 3800 Australia; c School of Mathematics, Statistics and Physics, Newcastle University Herschel Building Newcastle upon Tyne NE1 7RU UK; d School of Chemistry, University of Glasgow, University Pl Glasgow G12 8QQ UK Pablo.docampo@glasgow.ac.uk; e Optoelectronic Materials Section, Department of Chemical Engineering, Delft University of Technology Julianalaan 136 2628 BL Delft The Netherlands; f Lehrstuhl für Funktionelle Materialien, Physik-Department, Technische Universität München James-Franck-Str. 1 85748 Garching Germany; g Heinz Maier-Leibnitz Zentrum (MLZ), Technische Universität München Lichtenbergstr. 1 85748 Garching Germany

## Abstract

A better understanding of the materials' fundamental physical processes is necessary to push hybrid perovskite photovoltaic devices towards their theoretical limits. The role of the perovskite grain boundaries is essential to optimise the system thoroughly. The influence of the perovskite grain size and crystal orientation on physical properties and their resulting photovoltaic performance is examined. We develop a novel, straightforward synthesis approach that yields crystals of a similar size but allows the tuning of their orientation to either the (200) or (002) facet alignment parallel to the substrate by manipulating dimethyl sulfoxide (DMSO) and tetrahydrothiophene-1-oxide (THTO) ratios. This decouples crystal orientation from grain size, allowing the study of charge carrier mobility, found to be improved with larger grain sizes, highlighting the importance of minimising crystal disorder to achieve efficient devices. However, devices incorporating crystals with the (200) facet exhibit an s-shape in the current density–voltage curve when standard scan rates are used, which typically signals an energetic interfacial barrier. Using the drift-diffusion simulations, we attribute this to slower-moving ions (mobility of 0.37 × 10^−10^ cm^2^ V^−1^ s^−1^) in combination with a lower density of mobile ions. This counterintuitive result highlights that reducing ion migration does not necessarily minimise hysteresis.

## Abstract 2

Grain size and orientation's impact on charge carriers is explored *via* a new solvent engineering method for MAPbI_3_ solar cells. Drift-diffusion simulations connect s-shaped *JV* curves to slower ions.

## Graphical abstract

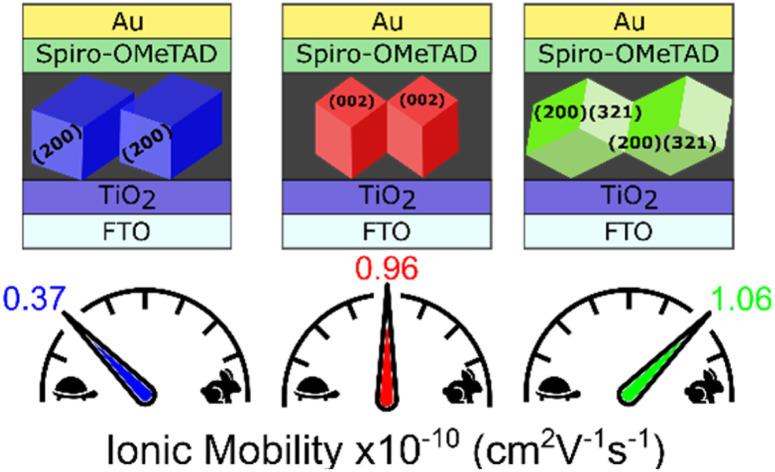

## Introduction

Perovskite solar cells have undergone extraordinary development within only a decade. The highest certified single-junction device performance exceeds 25% making the material a respectable competitor against or, in the case of tandem devices, ally with silicon solar cells.^1,2^ The exact parameters required for highly efficient perovskite photovoltaic devices remain debated. More so, a question arises as to which extent perovskite crystallinity and specific crystallographic alignment of the perovskite grains affect the device efficiency.

With the superior defect physics in hybrid lead-based perovskite materials, grain boundaries seem to become relatively insignificant and non-radiative recombination is more pronounced at the interfaces than in bulk.^3^ Thus, the highest efficiencies, as is the case for the 25% record, are achieved with polycrystalline mixed cation and anion perovskite-based absorber layers.^1–4^ The wider bandgap perovskite methylammonium lead iodide (MAPbI_3_) typically performs less efficiently in photovoltaics, with reported record efficiencies of 21% for single-crystalline MAPbI_3_-based devices.^4,5^ Similar high device efficiencies with polycrystalline thin MAPbI_3_ films only resulted from perovskite grain sizes in the micrometre to millimetre ranges with preferred perovskite crystal alignment in one direction.^6^ Devices incorporating smaller and less ordered MAPbI_3_ grains reach slightly lower efficiencies.^7–9^ However, it is not entirely clear whether improving crystal size and alignment in one direction is the objective for further enhancement of polycrystalline perovskite-based device performance with the highest potential or whether the specific crystal alignment directions have an additional impact. We note here that an appreciation of the crystallisation of triple cation perovskite devices is also important; however, due to the continued popularity of MAPbI_3_-based devices this study will focus solely on this class of material.

Although the impact of perovskite grain boundaries on the optoelectronic performance of the material is small with polycrystalline films, especially with passivation, limitations apply to charge transport through the borders.^10,11^ Additionally, bulk defect heterogeneities from different grains are evident in photoluminescence and cathodoluminescence investigations of polycrystalline perovskite films.^12–14^ Studies on other perovskite single-crystal facets also confirm a difference in trap and ion densities at varying crystal facets.^15–17^ Ion mobility is a crucial parameter in perovskite-based devices. Rather than trapping the charges, the defects formed at grain boundaries seem to assist ion migration in the perovskite films, dictating the overall solar cell performance and stability.^18^ Mobile ions negatively affect perovskite device stability, such as inducing phase segregation^19^ or negatively accumulated ions at the interface leading to energetical barriers;^20^ encapsulation does not avoid this type of degradation.^21,22^ The ion movement also influences anomalies in current density–voltage (*J–V*) measurements, mainly observed as hysteresis in perovskite-based devices.^23^ Ion mobility is linked to the Coulomb interaction between crystal planes and the mobile ions, and hence, modification of such crystallographic planes of the perovskite crystal can show the exact impact of certain crystal facets at the interfaces to the charge transport layers and between grain boundaries; control over this could further improve perovskite solar cell performance.^24^

In this work, we take a closer look at the influence of the amount and the nature of perovskite grain boundaries arising from grain size and orientation. We developed a thin-film synthesis procedure that allows us to vary the grain size and crystal alignment while retaining similar deposition conditions. Using time-resolved microwave conductivity (TRMC) and time-of-flight (ToF) transient photoconductivity, we attribute an improvement in charge carrier mobility to increased grain sizes and degree of crystal alignment in the films but not to one specific crystal orientation direction. However, in devices, the *J–V* curves measured show a highly pronounced s-shape and overall lower performance if perovskite films consisting of the largest grains and perfect alignment in one direction are applied in combination with a TiO_2_ or SnO_2_ electron charge transport layer. We show that this s-shape is a result of a combination of the scan rate used and reduced ion mobility in these films. Furthermore, the s-shape can be removed by employing organic interface materials such as PCBM, C_60_ or PEDOT:PSS which can compensate for the detrimental effects of ion accumulation at the interface by introducing an energy offset or passivating the perovskite surface. More importantly, the devices implementing the largest and most highly ordered crystallites with organic interfaces have the highest performance and higher charge mobilities.

## Results & discussion

The fabrication of perovskite thin films with varying degrees and directions of crystal alignment usually requires significant changes in the synthesis procedures.^25^ Different fabrication conditions can alter the defect formation, grain size orientation, *etc.* Here, we demonstrate a synthesis approach to overcome these challenges by only introducing up to 20 vol% of the solvent additive dimethyl sulfoxide (DMSO) to a Pb-acetate-based precursor solution. The amount of DMSO additive in specific precursor solution concentrations changes the crystal alignment dramatically from highly disordered polycrystalline thin films to highly aligned systems with the long *c*-axis of the tetragonal crystals perpendicular or parallel to the substrate. Moreover, we can tune the grain size while maintaining the perfect alignment in one direction by exchanging the DMSO additive with tetrahydrothiophene-1-oxide (THTO). Effectively, our approach allows control over crystal orientation, grain size and other morphology-related differences, allowing us to study its influence on the resulting devices.

**Fig. 1:**
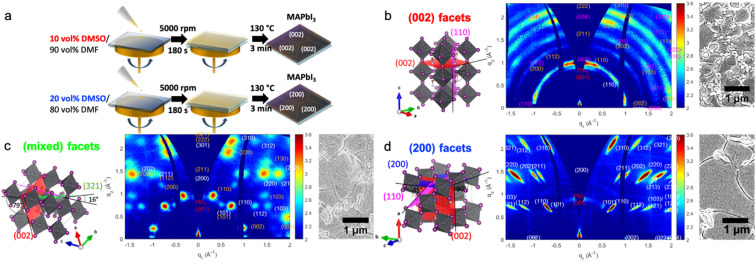
(a) Schematic illustration of the synthesis process with varying DMSO concentrations to achieve either (002) or (200) facet alignment in a precursor solution containing 1.875 mmol mL^−1^ PbAc_2_ and 5.625 mmol mL^−1^ MAI. (b–d) Schematic illustrations (left) of the different crystal facets parallel to the substrate, representing the main orientation deducted from 2D GIWAXS data (middle) and the SEM images of the respective films (right). (b) Perovskite film with the (002) facet parallel to the substrate; (c) perovskite film with (200) and (321) facets parallel to the substrate; (d) perovskite film with the (200) facet parallel to the substrate.

We attribute the change in crystal orientation to the stabilisation of different low-dimensional non-perovskite precursor phases through the solvent additives. These precursor phases can either originate from the lead acetate precursor or DMSO.^26,27^ Thus, the crystal alignment in the perovskite films of our study strongly depends on which of the two precursor phases dominates during the layer deposition. In short, our previous report shows that a strongly stabilised lead acetate-based precursor phase results in perfectly oriented (200) facets of the resulting tetragonal perovskite crystals.^26^ To achieve this alignment, our experiments required using an equal molar ratio between the lead precursor and DMSO additive or excess DMSO up to double the amount compared to lead. Perovskite films with the preferred (002) facet alignment formed using less or more DMSO additive than necessary for the perovskite films with (200) facets. The typical complex of perovskite with DMSO has the stoichiometric formula MA_2_Pb_3_I_8_(DMSO)_2_ typically resulting in a highly preferred crystal alignment of the (110) perovskite facet parallel to the substrate even with a high molar excess of DMSO compared to lead in the precursor solution.^27–29^ However, in this study, we obtain the preferred crystal alignment of the (002) facet parallel to the substrate instead of the similar crystal lattice distanced (110).^16^Fig. 1a and the ESI† give detailed synthesis conditions.

To analyse the type and degree of perovskite crystal alignment concerning the substrate, we performed grazing-incidence wide-angle X-ray scattering (GIWAXS) experiments. This technique captures a two-dimensional slice through reciprocal space, reconstructing the structure and extracting information on the crystal planes' orientation from the azimuthal intensity distribution.^30–33^ Hence, with the GIWAXS data shown in Fig. 1, we can confirm the synthesis of perovskite films with strong alignment parallel to the substrate of the (002) facet. Here, the perovskite crystals stand with their *c*-axis perpendicular to the substrate 10 vol% DMSO in a 62 wt% precursor solution in dimethyl formamide (DMF). Changing to an approximately equal molar ratio of lead and DMSO additive in the precursor solution using 15 vol% DMSO in a 62 wt% precursor solution, we identify a mixture of two facets, hereon termed mixed facets; about half of the crystals are present in the (200) orientation while the second orientation is the best corresponding to the (312) crystal plane, parallel to the substrate. The pure alignment of the (200) crystal facet parallel to the substrate, hereon termed the (200) facet, results from a similar precursor solution with a slight excess in the molar ratio of DMSO to lead using 20 vol% DMSO. No preference in crystal alignment results from pure DMF-based solutions (Fig. S1d†). To further investigate orientations in the various films produced, contrast-enhanced intensity mapping of the GIWAX measurements was performed, the results of which are displayed in Fig. S2.† For the films formed from 10 vol% and 20 vol% DMSO, the dominance of the (002) and (200) peaks respectively suggest a near-perfect alignment of the crystal facets to the substrate, and therefore a percentage close to 100%. The film formed from 15 vol% DMSO shows two dominant phases. Their ratio was determined by comparing their respective intensities, extracted from a pixel-intensity map analysis of their peaks. Here, the (200) facet was found to correspond to 57.8% of the mixed-phase system, with the remaining 42.2% corresponding to the (312) orientation.

We note here that the crystal alignment direction and degree of variation are highly reproducible and are confirmed with GIWAXS and XRD studies (see Fig. S3†). The results were reproduced in two different labs (in Germany and Australia) with slight adjustments to the synthesis protocol described in the ESI.† This indicates that the crystal orientation in the final perovskite film deposited *via* the one-step method results from the precursor species' coordination chemistry and the orientation of intermediate phases.^34^

On the left of Fig. 1b–d, we show the schematics of the three main differences in the perovskite facet alignments studied here. The scanning electron microscopy (SEM) top views on the right in Fig. 1b–d and the images of the thin film cross-sections in Fig. S1a–c (ESI)† show that the morphology of the films remains similar with a slight increase in grain size with a higher amount of DMSO in the precursor solution. The thin film thickness defined by a single grain is, in all cases, similar. When using the THTO additive instead of DMSO, we have approximately tripled the sizes of the grains aligned with the (200) facet parallel to the substrate, as shown in our previous work and some additional SEM analyses in ESI Fig. S1f†.^26^

To study if the differences in the number and nature of grain boundaries in the lateral direction result in changed physical properties, we investigate their charge carrier mobilities with time-of-flight transient photoconductivity (ToF). ToF allows the determination of charge carrier mobility upon pulsed laser excitation; the experimental setup can be seen in Fig. S4a.† To this end, we employ a lateral sample layout with the twelve electrode pairs spaced (d) at varying distances between 20 and 80 μm (shown in Fig. S4b†), which allows us to perform photocurrent measurements at constant applied electric fields *E* (here: 5 kV cm^−1^). Illuminating the perovskite film directly (excitation with a 510 nm laser) in contact with the electrodes at the edge of one contact creates charge carriers locally (an approximate area of 13 μm^2^ gives a laser spot size of 2 μm) and extracts them at the opposite electrode with a lateral distance *d*, as shown in Fig. S4c.† Hence, the applied field's polarity determines the type of charges probed (positive or negative charges, hereon termed as holes and electrons). The obtained transients, as shown in an example in ESI Fig. S3d,† are plotted on a double log scale while the intersection of the pre-and post-transit defines the transit time *t*_tr_, which is needed to calculate the mobility according to:1
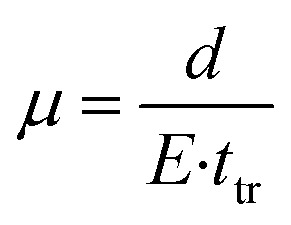


**Fig. 2:**
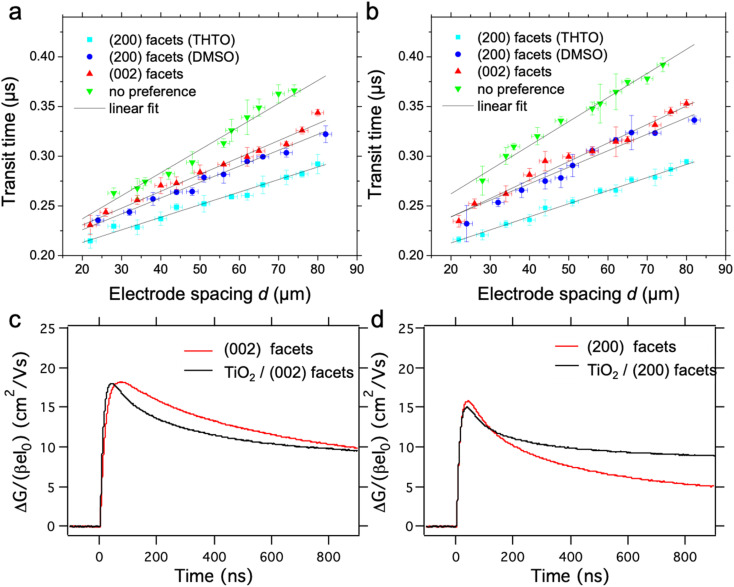
(a and b) ToF analysis data of perovskite films prepared from different solvent additive concentrations: (a) extracted transit times for electrons from ToF analysis; (b) extracted transit times for holes from ToF; (c and d) time-resolved microwave conductance (TRMC) traces recorded with an incident intensity of 2.3 × 10^10^ photons cm^−2^ with a 10 Hz repetition rate (*λ* = 650) for MAPbI_3_ with the (002) facet (c) and (200) facet (d) on quartz (red) and planar TiO_2_ (black).

A detailed description of the ToF setup and the results obtained from the measurement can be found in Fig. 2a, b and the ESI.†Table 1 summarises the extracted charge carrier mobility values of films with no preference for crystal alignment and the high order in each of the two distinct directions. The grain sizes are only slightly smaller for the perovskite films showing preferred crystal alignment with (002) facets compared to the films with a high degree of order with the (200) facets, as indicated in the SEM top-view images in Fig. 1b, d and S1f.† However, the charge carrier mobility extracted from ToF analysis is very similar for DMSO-based (002) and (200) facet films. Smaller values (reduced by 3 cm^2^ V^−1^ s^−1^) are deducted from perovskite films with no preferential crystal alignment with similar grain sizes as the (002) facet-based films, as shown in Fig. S1e.† Therefore, we attribute a positive impact on electron- or hole-carrier mobility to the improved degree in crystal order of (002) and (200) facet samples, but no differences appear for the charge carrier mobilities crossing the two distinct types of grain boundaries. With larger grain sizes (more than double on average) and similar crystal alignment, the charge carrier mobilities further improve by around 3 cm^2^ V^−1^ s^−1 26^. However, the perfect alignment of the perovskite crystallites only corresponds to the out-of-plane direction and not the lateral direction to which our ToF setup is limited.

**Table 1:** The extracted electron- and hole-mobility from ToF measurements

Crystal facet alignment	Electron mobility/cm^2^ V^−1^ s^−1^	Hole mobility/cm^2^ V^−1^ s^−1^	Standard deviation
(200) THTO	16	15	1
(200) DMSO	13	12	1
(002)	12	11	1
No. pref	9	8	1

Time-resolved microwave conductivity (TRMC) enables the investigation of the local perovskite charge carrier mobilities in the in-plane direction with local information on charge transport throughout the whole sample without the need for electrical contacts. TRMC is a powerful method to study the dynamics of photoinduced charge carriers, based on the interactions between the electric field component of microwaves and mobile carriers.^35^ In Fig. 2c and d, intensity-normalized photoconductance transients are shown for both ((200) and (002)) orientations deposited on quartz (red) or TiO_2_ (black) on excitation at 650 nm. Typically, on pulsed excitation, the photoconductance increases sharply as a result of the photo generation of mobile charge carriers, which is followed by their decay due to charge recombination and/or immobilisation of charge carriers in trap states. We observe maximum mobilities between 15 and 20 cm^2^ V^−1^ s^−1^ for both orientations deposited on a quartz substrate, comparable with previously reported values.^36^ Although the obtained values are higher than the ones obtained by ToF, both techniques clearly show that the direction of crystal orientation has no impact on charge carrier mobility if measured in the direction perpendicular to the ordered facets. With TRMC, we observe a gradual reduction in charge carrier lifetime and signal height on increasing the laser intensity, which is characteristic of higher-order recombination.^36^ Under similar excitation conditions, the lifetime is somewhat shorter for the (200) orientation. In a bilayer combination of MAPbI_3_ on top of TiO_2,_ our TRMC results in Fig. 2 suggest an inefficient electron transfer for both bilayer combinations with (200) or (002) facets. Thus, our TRMC results indicate no heterogeneities for charge transfer for different perovskite facets at the interface. These results are analogous to previously reported MAPbI_3_/TiO_2_ bilayer systems.^37^ In the case of efficient electron transfer into TiO_2_, we expect that the TRMC signal lowers since the excess electrons in TiO_2_ have low mobility and will hardly contribute to the photoconductance as has been reported.^38,39^ The anomaly to this in Fig. 2d shows that there is a higher mobility after 200 ns for the (200) facet on TiO_2_. This is likely due to poorer electron extraction which is investigated in the discussion below. We note here that surface defects are likely to have a greater effect on the charge carrier lifetime and mobility that on the grain orientation alone.^40^ However, since the same perovskite system is used in comparison of experimental and simulated techniques we focus on the effects of the grain size and orientation solely.

**Fig. 3:**
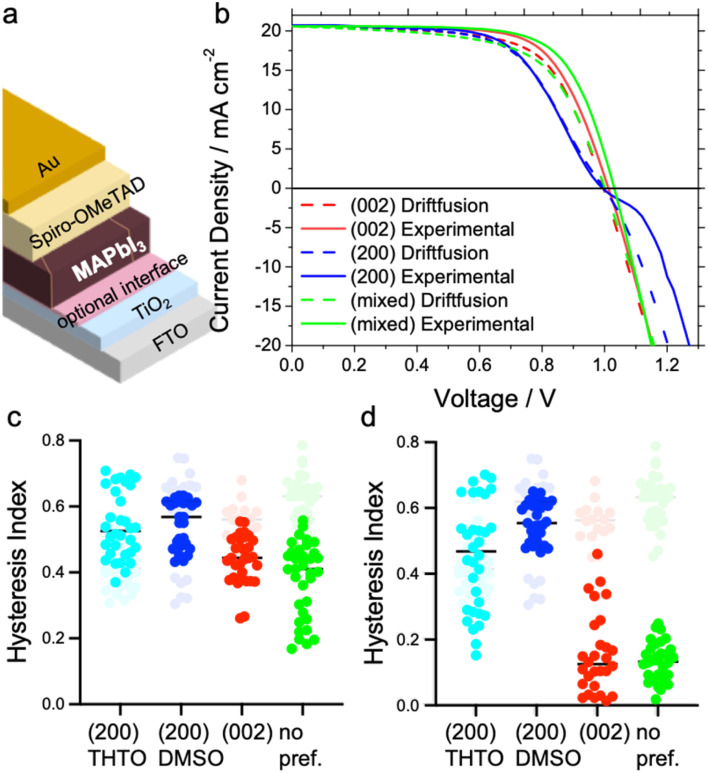
(a) Schematic illustration of the solar cell device architecture. (b) *J*–*V* curves: solid lines represent the experimental results from devices with the architecture from (a) without the optional interface and the varying perovskite crystal alignment preferences. Dashed lines represent the simulation results from Driftfusion. A low ionic mobility of 0.37 × 10^−10^ cm^2^ V^−1^ s^−1^ was used to fit the (200) facet perovskite, which showed s-shape behaviour, whilst higher ion mobilities of 0.96 × 10^−10^ cm^2^ V^−1^ s^−1^ and 1.06 × 10^−10^ cm^2^ V^−1^ s^−1^ were used to simulate the (002) and (200) and (321) facet perovskites, respectively; (c and d) hysteresis index of solar cell devices with an architecture as indicated in (a) with varying perovskite crystal alignments; the graphs in grey indicate the results from devices with only TiO_2_ at the interface with the perovskite, and those in colour, devices with an additional PCBM interface with a thin layer in (c) and thick layer in (d).

To study the impact of the amount and nature of grain boundaries on the photovoltaic performance, we prepare devices in the regular architecture with the perovskite layers sandwiched between TiO_2_ and Spiro-OMeTAD as displayed in Fig. 3a. Typically, perovskite-based devices with this architecture show very high *J–V* hysteresis on the forward and reverse scans. Our results show an additional anomaly in the *J–V* curve with devices perfectly aligned with the (200) facet parallel to the substrate, as shown in Fig. 3b. In particular, an s-kink exclusively appears in the *J–V* curves for all devices incorporating absorber layers with perfect alignment of the long perovskite *c*-axis parallel to the substrate, either with the DMSO or THTO additive (see Fig. 3b, S5a and S7a†). We note that the growth of the MAPbI_3_ films with the (200) facet from DMSO and THTO also results in larger grain sizes but, doubling the overall grain to around 10 μm has no effect on the s-shape of the *J–V* curves. The appearance of the s-kink mainly reduces the open-circuit voltage (*V*_OC_) and fill factor (FF) of the devices. A counterintuitive result is that the mixed facet orientation device (*i.e.* most disordered) displays the highest *V*_OC_ and FF (see Fig. 3b and S5a†).

The change in the SnO_2_ electron transport layer instead of TiO_2_ can help to decrease the hysteresis in perovskite-based solar cells.^41^ In our experiments, both the hysteresis and the appearance of the s-kink strictly related to (200) perovskite facet in devices remain unchanged if SnO_2_ or TiO_2_ is used as the charge transport layers (see Fig. S5c†). Only the addition of a C_60_ monolayer or a phenyl-C61-butyric acid methyl ester (PCBM) layer at the metal oxide/MAPbI_3_ interface avoids the appearance of the s-kink using the perovskite films with the (200) facets aligned (see Fig. S5c†). Therefore, the overall device efficiency improves, such that the (200) facet devices in this configuration show the highest performance at around 18.5% efficiency compared to the other perovskite films.^23^ Persistent hysteresis is observed in the (200) facet with the addition of the fullerene layers whereas in the (002) facet case hysteresis is diminished (Fig. 3c, d and S6†). In an inverted device configuration with poly(3,4-ethylenedioxythiophene) polystyrene sulfonate (PEDOT:PSS) and PCBM, the high-quality perovskite (200) facet films reveal the highest potential with fill factors of around 80% and the lowest hysteresis in the range of our experiments (see Fig. S5d, S6c and f†).

The hysteresis, as understood, is caused by the redistribution of mobile ion vacancies at the interfaces to the charge extraction layers screening the perovskite's internal electric field.^23,42–44^ According to simulations, *J–V* hysteresis vanishes in the presence of mobile ions if the surface recombination at the perovskite interface to the charge extraction layers is low and the charge carrier diffusion length in the perovskite film is high.^45^ Therefore, the choice of charge transport material can affect the *J–V* hysteresis.^23,43,46,47^ The disappearance of the s-shape in all of our *J–V* curves and reduction of hysteresis with organic interfaces sandwiching the perovskite layer is in line with reported single-crystalline MAPbI_3_ devices.^4,5,48^ In the study by Chen *et al.*, the single-crystal films were grown with the (200) facet parallel to the substrate, similar to our highest crystalline and ordered layers prone to s-shapes of the *J–V* curves in devices. In their study, the single-crystal growth protocol required using substrates covered with poly(triaryl amine) PTAA due to an ion diffusion constant compared to the high surface energy substrates like glass or ITO resulting in the continuous growth of the single-crystal layer.^48^ Thus, PTAA acted as a promoter for ionic transport during crystal growth and most likely during the solar cell device operation.^49^ Studies on ion migration in perovskite-based devices reached a similar conclusion that the organic interface material accelerates ion migration at the interface avoiding charge accumulation.^23,47^

To confirm the effect of ion mobility on the shape of the *J–V* curve, we perform a computational study using the Driftfusion software package.^44^ Using all the experimentally extracted materials' optoelectronic values, such as mobility, thickness, *etc.*, as inputs for the model, we show that the s-shape in Fig. 3b can be reproduced with a low ion mobility of approximately 0.37 × 10^−10 12^ cm^2^ V^−1^ s^−1^. Indeed, as the ion mobility increases beyond 0.5 × 10^−10 12^ cm^2^ V^−1^ s^−1^, the s-shape disappears, as shown in Fig. S11.† These ion-mobility values generally rank in the lower range of the reported values spanning from 10^−6^ to 10^−12^ cm^2^ V^−1^ s^−1^.^42,50–53^ Experimentally the ionic concentration can be determined through transient measurements such as time-resolved KPFM^54^ or step-dwell-step-probe measurements;^55^ however, exact determination of this quantity for our devices is out of the scope of this study. Here, ion mobility controls how ions behave at an applied voltage. With lower ion mobility, ions within the perovskite will accumulate less at the perovskite/ETL boundary under a forward bias than those with higher mobility (Fig. S8a†). This change in ionic redistribution controls the conduction band energy shape (Fig. S8b†), electrostatic potential profile (Fig. S9c†) and the local concentration of electrons and holes within the perovskite layer. Driftfusion simulations find that in the region of the *J–V* curve where the s-shape appears, there is a greater density of electrons at the perovskite/HTM boundary and a greater density of holes at the perovskite/ETL boundary for a simulated perovskite solar cell with lower ion mobility (Fig. S9a and b†). This leads to a greater interfacial recombination rate of charge carriers (Fig. S9d†), reducing the overall current from the solar cell and forming an s-shaped *J–V* curve. Effectively, we find that ions control the electronic charge transfer rate across perovskite interfaces, as Moia *et al.* demonstrated with a perovskite solar cell modelled as a bipolar junction transistor where ionic contributions are correlated with the base current.^56^ Ultimately, however, the role of scan rate cannot be underestimated. In our experimental results, measurements of our devices at 0.2 V s^−1^ show pronounced s-shaped *J*–*V* curves in the (200) facet devices. Our simulations further indicate that at slower scan rates for this device (with a predicted ion mobility of 0.37 × 10^−10^ cm^2^ V^−1^ s^−1^) s-shaped *J*–*V* curves are not observed, but on increasing the scan rate beyond 0.2 V s^−1^ much more pronounced s-shaped *J*–*V* curves are seen (Fig. S10†). For the device with the (002) orientated facet with a larger ionic mobility of 0.96 × 10^−10^ cm^2^ V^−1^ s^−1^, a different scan rate dependence is observed; both increasing and decreasing the scan rate from 0.2 V s^−1^ leads modification of the shape of the *J*–*V* curves in detrimental ways, indicating that for this device a scan rate of 0.2 V s^−1^ achieves the highest fill factor. This further corroborates the role of ions in controlling the *J*–*V* curve shape and the importance of the scan rate with mobile ions present.^44,57,58^

The findings from Driftfusion simulations are consistent with our device results. Here, an increased grain size leads to fewer grain boundaries, so defect formation is minimised, resulting in reduced ion mobility. Additionally, the nature of the grain boundaries seems to influence ionic motion greatly, with the general consensus in the literature suggesting that ionic motion is faster in grain boundaries than in the bulk itself^18,59–61^ pointing to minimizing the quantity of grain boundaries^59^ or passivation^60,62^ of this physical feature to increase the activation energy of mobile ions.^59^ Naturally, the increased density of defects in these regions is linked to the intrinsic stability of the perovskite. There is still no clear consensus on whether grain boundaries are beneficial or detrimental to the long-term stability of perovskite solar cells with some research suggesting that the electrical response of the grain boundaries can cause degradation to the pristine perovskite^63^ as well as the grain boundaries being suggested as the origin of thermal and moisture degradation *via* a process of separation of the bulk grains hindering optoelectronic performance and creating highways for moisture travel.^64^ On the other hand, other studies suggest that grain boundaries possess a similar photo-response compared to the bulk grains and hence are not the nucleation sites for degradation mechanisms.^65^ In some particular cases, with careful control of the grain boundary angle, moisture ingress and degradation channels can be suppressed.^66^ Ultimately, however, the morphology and composition of the grain boundaries are going to control the extent to which the various degradation pathways and ion migration channels affect the performance and stability of perovskite devices. In our experiments, specific devices with the (200) perovskite facets parallel to the substrates indicated the lowest ion mobilities in directions parallel to the crystal planes. In reported experiments comparing aligned (ribbon) films and non-ordered films with confocal photoluminescence microscopy, more uniform ionic transport was observed in the aligned (ribbon) films.^67^ These ribbons have crystal alignment comparable to our (002) perovskite facet films, pointing towards anisotropic ion motion depending on the perovskite crystal plane. These results also agree with the findings of anisotropy in charge carrier mobilities in varying perovskite crystal facets.^68^ Importantly, the route to control the grain size and orientation presented in this work is key to allowing further development and optimization of performance and stability relating to grain boundaries.

## Conclusions

All in all, we have developed a straightforward synthetic approach that allows targeting a specific perovskite crystal orientation and size in polycrystalline films by manipulating THTO and DMSO solvent ratios, respectively. Through this approach we are able to synthesize perovskite solution-processed films containing crystals well in excess of 30 μm while we are able to target perfectly oriented crystals in the (200) orientation or the (002) orientation, making this approach useful for applications where crystal orientation is important such as in optics applications. Furthermore, our approach leads to the minimisation of crystal disorder at grain boundaries which leads to a clear improvement in charge carrier mobility and device performance while simultaneously reducing ion mobility. We validate this result using two complementary techniques, time of flight and microwave conductivity measurements which place the mobility of our developed films at about 18 cm^2^ V^−1^ s^−1^, approximately twice what is achieved for disordered films. Our results show no indications of dependence on the crystal orientation direction for charge carriers' movement but we observe that ion diffusion is significantly affected depending on the orientation of the crystals. When incorporated into perovskite solar cells, our results show the appearance of s-shaped *J*–*V* curves for devices composed of crystals oriented with the long axis parallel to the substrate, *i.e.* (002). This s-shape seemingly disappears when employing organic materials as the charge extraction contacts instead of metal oxides, *i.e.* SnO_2_ and TiO_2_. Overall, our approach demonstrates that minimising disorder at the perovskite grain boundaries is an effective route to maximise charge transport in perovskite films and leads to clear improvements in device performance. Furthermore, our results challenge the pervasive notion in the field that minimising ion migration is an effective route to eliminate hysteresis. Indeed, our results conclusively show that minimising ion migration may exacerbate hysteresis and yield anomalous results, particularly when experiments are performed at scan rates that correlate with the timescales of ion migration.

## Conflicts of interest

There are no conflicts to declare.

## Supplementary Material

TA-012-D3TA04458K-s001

## References

[cit1] NREL, Best Research-Cell Efficiency Chart, https://www.nrel.gov/pv/cell-efficiency.html, accessed 7 May 2023

[cit2] Yoo J. J., Seo G., Chua M. R., Park T. G., Lu Y., Rotermund F., Kim Y.-K., Moon C. S., Jeon N. J., Correa-Baena J.-P., Bulović V., Shin S. S., Bawendi M. G., Seo J. (2021). Nature.

[cit3] Yang Y., Yang M., Moore D. T., Yan Y., Miller E. M., Zhu K., Beard M. C. (2017). Nat. Energy.

[cit4] Chen Z., Turedi B., Alsalloum A. Y., Yang C., Zheng X., Gereige I., AlSaggaf A., Mohammed O. F., Bakr O. M. (2019). ACS Energy Lett..

[cit5] Alsalloum A. Y., Turedi B., Zheng X., Mitra S., Zhumekenov A. A., Lee K. J., Maity P., Gereige I., AlSaggaf A., Roqan I. S., Mohammed O. F., Bakr O. M. (2020). ACS Energy Lett..

[cit6] Fan H., Li F., Wang P., Gu Z., Huang J.-H., Jiang K.-J., Guan B., Yang L.-M., Zhou X., Song Y. (2020). Nat. Commun..

[cit7] Ahn N., Son D.-Y., Jang I.-H., Kang S. M., Choi M., Park N.-G. (2015). J. Am. Chem. Soc..

[cit8] Wu Y., Xie F., Chen H., Yang X., Su H., Cai M., Zhou Z., Noda T., Han L. (2017). Adv. Mater..

[cit9] Li J., Dagar J., Shargaieva O., Flatken M. A., Köbler H., Fenske M., Schultz C., Stegemann B., Just J., Többens D. M., Abate A., Munir R., Unger E. (2021). Adv. Energy Mater..

[cit10] Reid O. G., Yang M., Kopidakis N., Zhu K., Rumbles G. (2016). ACS Energy Lett..

[cit11] Adhyaksa G. W. P., Brittman S., Āboliņš H., Lof A., Li X., Keelor J. D., Luo Y., Duevski T., Heeren R. M. A., Ellis S. R., Fenning D. P., Garnett E. C. (2018). Adv. Mater..

[cit12] Bischak C. G., Sanehira E. M., Precht J. T., Luther J. M., Ginsberg N. S. (2015). Nano Lett..

[cit13] de Quilettes D. W., Vorpahl S. M., Stranks S. D., Nagaoka H., Eperon G. E., Ziffer M. E., Snaith H. J., Ginger D. S. (2015). Science.

[cit14] Leblebici S. Y., Leppert L., Li Y., Reyes-Lillo S. E., Wickenburg S., Wong E., Lee J., Melli M., Ziegler D., Angell D. K., Ogletree D. F., Ashby P. D., Toma F. M., Neaton J. B., Sharp I. D., Weber-Bargioni A. (2016). Nat. Energy.

[cit15] Kim D., Yun J.-H., Lyu M., Kim J., Lim S., Yun J. S., Wang L., Seidel J. (2019). J. Phys. Chem. C.

[cit16] Ding J., Jing L., Yuan Y., Zhang J., Yao Q., Wang K., Zhang W., Sun H., Liu B., Zhou T., Zhan X. (2020). ACS Appl. Energy Mater..

[cit17] Zuo Z., Ding J., Li Y., Zhao Y., Du S. (2018). Mater. Res. Bull..

[cit18] Shao Y., Fang Y., Li T., Wang Q., Dong Q., Deng Y., Yuan Y., Wei H., Wang M., Gruverman A., Shield J., Huang J. (2016). Energy Environ. Sci..

[cit19] Zhang D., Li D., Hu Y., Mei A., Han H. (2022). Commun. Mater..

[cit20] Ebadi F., Taghavinia N., Mohammadpour R., Hagfeldt A., Tress W. (2019). Nat. Commun..

[cit21] Li X., Wang X., Zhang W., Wu Y., Gao F., Fang J. (2015). Org. Electron..

[cit22] Berry J., Buonassisi T., Egger D. A., Hodes G., Kronik L., Loo Y.-L., Lubomirsky I., Marder S. R., Mastai Y., Miller J. S., Mitzi D. B., Paz Y., Rappe A. M., Riess I., Rybtchinski B., Stafsudd O., Stevanovic V., Toney M. F., Zitoun D., Kahn A., Ginley D., Cahen D. (2015). Adv. Mater..

[cit23] Richardson G., O'Kane S. E. J., Niemann R. G., Peltola T. A., Foster J. M., Cameron P. J., Walker A. B. (2016). Energy Environ. Sci..

[cit24] Zai H., Ma Y., Chen Q., Zhou H. (2021). J. Energy Chem..

[cit25] Oesinghaus L., Schlipf J., Giesbrecht N., Song L., Hu Y., Bein T., Docampo P., Müller-Buschbaum P. (2016). Adv. Mater. Interfaces.

[cit26] Giesbrecht N., Schlipf J., Grill I., Rieder P., Dyakonov V., Bein T., Hartschuh A., Müller-Buschbaum P., Docampo P. (2018). J. Mater. Chem. A.

[cit27] Bai Y., Xiao S., Hu C., Zhang T., Meng X., Li Q., Yang Y., Wong K. S., Chen H., Yang S. (2017). Nano Energy.

[cit28] Chen H., Ding X., Xu P., Hayat T., Alsaedi A., Yao J., Ding Y., Dai S. (2018). ACS Appl. Mater. Interfaces.

[cit29] Cao J., Jing X., Yan J., Hu C., Chen R., Yin J., Li J., Zheng N. (2016). J. Am. Chem. Soc..

[cit30] Saliba M., Tan K. W., Sai H., Moore D. T., Scott T., Zhang W., Estroff L. A., Wiesner U., Snaith H. J. (2014). J. Phys. Chem. C.

[cit31] Giesbrecht N., Schlipf J., Oesinghaus L., Binek A., Bein T., Müller-Buschbaum P., Docampo P. (2016). ACS Energy Lett..

[cit32] Müller-Buschbaum P. (2014). Adv. Mater..

[cit33] Schlipf J., Müller-Buschbaum P. (2017). Adv. Energy Mater..

[cit34] Rahimnejad S., Kovalenko A., Forés S. M., Aranda C., Guerrero A. (2016). ChemPhysChem.

[cit35] Savenije T. J., Guo D., Caselli V. M., Hutter E. M. (2020). Adv. Energy Mater..

[cit36] Hutter E. M., Eperon G. E., Stranks S. D., Savenije T. J. (2015). J. Phys. Chem. Lett..

[cit37] Wojciechowski K., Stranks S. D., Abate A., Sadoughi G., Sadhanala A., Kopidakis N., Rumbles G., Li C.-Z., Friend R. H., Jen A. K.-Y., Snaith H. J. (2014). ACS Nano.

[cit38] Hutter E. M., Hofman J.-J., Petrus M. L., Moes M., Abellón R. D., Docampo P., Savenije T. J. (2017). Adv. Energy Mater..

[cit39] Ponseca C. S., Savenije T. J., Abdellah M., Zheng K., Yartsev A., Pascher T., Harlang T., Chabera P., Pullerits T., Stepanov A., Wolf J.-P., Sundström V. (2014). J. Am. Chem. Soc..

[cit40] Yang Y., Yang M., Moore D. (2017). Nat. Energy.

[cit41] Aygüler M. F., Hufnagel A. G., Rieder P., Wussler M., Jaegermann W., Bein T., Dyakonov V., Petrus M. L., Baumann A., Docampo P. (2018). ACS Appl. Mater. Interfaces.

[cit42] van Reenen S., Kemerink M., Snaith H. J. (2015). J. Phys. Chem. Lett..

[cit43] Kim H.-S., Jang I.-H., Ahn N., Choi M., Guerrero A., Bisquert J., Park N.-G. (2015). J. Phys. Chem. Lett..

[cit44] Calado P., Telford A. M., Bryant D., Li X., Nelson J., O'Regan B. C., Barnes P. R. F. (2016). Nat. Commun..

[cit45] Neukom M. T., Schiller A., Züfle S., Knapp E., Ávila J., Pérez-del-Rey D., Dreessen C., Zanoni K. P. S., Sessolo M., Bolink H. J., Ruhstaller B. (2019). ACS Appl. Mater. Interfaces.

[cit46] DuBose J. T., Kamat P. V. (2020). J. Am. Chem. Soc..

[cit47] Courtier N. E., Cave J. M., Foster J. M., Walker A. B., Richardson G. (2019). Energy Environ. Sci..

[cit48] Chen Z., Dong Q., Liu Y., Bao C., Fang Y., Lin Y., Tang S., Wang Q., Xiao X., Bai Y., Deng Y., Huang J. (2017). Nat. Commun..

[cit49] Sendner C., Horinek D., Bocquet L., Netz R. R. (2009). Langmuir.

[cit50] Eames C., Frost J. M., Barnes P. R. F., O'Regan B. C., Walsh A., Islam M. S. (2015). Nat. Commun..

[cit51] Delugas P., Caddeo C., Filippetti A., Mattoni A. (2016). J. Phys. Chem. Lett..

[cit52] Birkhold S. T., Precht J. T., Giridharagopal R., Eperon G. E., Schmidt-Mende L., Ginger D. S. (2018). J. Phys. Chem. C.

[cit53] Yuan Y., Chae J., Shao Y., Wang Q., Xiao Z., Centrone A., Huang J. (2015). Adv. Energy Mater..

[cit54] Weber S. A. L., Hermes I. M., Turren-Cruz S. H., Gort C., Bergmann V. W., Gilson L., Hagfeldt A., Graetzel M., Tress W., Berger R. (2018). Energy Environ. Sci..

[cit55] Bertoluzzi L., Belisle R. A., Bush K. A., Cheacharoen R., McGehee M. D., O'Regan B. C. (2018). J. Am. Chem. Soc..

[cit56] Moia D., Gelmetti I., Calado P., Fisher W., Stringer M., Game O., Hu Y., Docampo P., Lidzey D., Palomares E., Nelson J., Barnes P. R. F. (2019). Energy Environ. Sci..

[cit57] Tress W., Marinova N., Moehl T., Zakeeruddin S. M., Nazeeruddin M. K., Grätzel M. (2015). Energy Environ. Sci..

[cit58] García-Rodríguez R., Riquelme A. J., Cowley M., Valadez-Villalobos K., Oskam G., Bennett L. J., Wolf M. J., Contreras-Bernal L., Cameron P. J., Walker A. B., Anta J. A. (2022). Energy Technol..

[cit59] McGovern L., Koschany I., Grimaldi G., Muscarella L. A., Ehrler B. (2021). J. Phys. Chem. Lett..

[cit60] Zhao Y., Zhou W., Han Z., Yu D., Zhao Q. (2021). Phys. Chem. Chem. Phys..

[cit61] Kim T., Park S., Iyer V., Shaheen B., Choudhry U., Jiang Q., Eichman G., Gnabasik R., Kelley K., Lawrie B., Zhu K., Liao B. (2023). Nat. Commun..

[cit62] Ma Y., Cheng Y., Xu X., Li M., Zhang C., Cheung S. H., Zeng Z., Shen D., Xie Y., Chiu K. L., Lin F., So S. K., Lee C. S., Tsang S. W. (2021). Adv. Funct. Mater..

[cit63] Hui Y., Tan Y.-Y., Chen L., Nan Z. A., Zhou J. Z., Yan J. W., Mao B. W. (2021). Adv. Funct. Mater..

[cit64] Castro-Méndez A. F., Hidalgo J., Correa-Baena J. P. (2019). Adv. Energy Mater..

[cit65] Chu Z., Yang M., Schulz P., Wu D., Ma X., Seifert E., Sun L., Li X., Zhu K., Lai K. (2017). Nat. Commun..

[cit66] Chun D. H., Kim S., Chai S. U., Kim W., Kim W., Lee J. H., Rhee R., Choi D., Kim J. K., Shin H., Park J. H. (2019). Nano Lett..

[cit67] Fassl P., Ternes S., Lami V., Zakharko Y., Heimfarth D., Hopkinson P. E., Paulus F., Taylor A. D., Zaumseil J., Vaynzof Y. (2019). ACS Appl. Mater. Interfaces.

[cit68] Meng G., Feng Y., Song X., Shi Y., Ji M., Xue Y., Hao C. (2018). J. Electroanal. Chem..

