# Supplementary material for: The balancing act between high electronic and low ionic transport influenced by perovskite grain boundaries
Source: J Mater Chem A Mater. 2024 Mar 21;12(19):11635–43. doi: 10.1039/d3ta04458k (PMC11093097; doi:10.1039/d3ta04458k)
Supplement: TA-012-D3TA04458K-s001 [file TA-012-D3TA04458K-s001.pdf]

Electronic Supplementary Material (ESI) for Journal of Materials Chemistry A.
This journal is © The Royal Society of Chemistry 2024

Supporting Information

The balancing act between high electronic and low ionic transport influenced by perovskite grain boundaries

Nadja Glück[1,2†], Nathan S Hill[3†], Marcin Giza[4], Eline Hutter[5], Irene Grill[1], Johannes Schlipf[6], Udo Bach[2], Peter Müller-Buschbaum[6,7], Achim Hartschuh[1], Thomas Bein[1], Tom Savenije[5], Pablo Docampo[4*]

[1] Department of Chemistry and Center for NanoScience (CeNS), University of Munich (LMU), Butenandtstr. 5-13, 81377 München, Germany.

[2] Department of Chemical Engineering, Monash University, Clayton, Victoria 3800 Australia

[3] School of Mathematics, Statistics and Physics, Newcastle University, Herschel Building, NE1 7RU, Newcastle upon Tyne, UK.

[4] Optoelectronic Materials Section, Department of Chemical Engineering, Delft University of Technology, Julianalaan 136, 2628 BL Delft, The Netherlands.

[5] Lehrstuhl für Funktionelle Materialien, Physik-Department, Technische Universität München, James-Franck-Str. 1, 85748 Garching, Germany.

[6] Heinz Maier-Leibnitz Zentrum (MLZ), Technische Universität München, Lichtenbergstr. 1, 85748 Garching, Germany

[7] School of Chemistry, University of Glasgow, University Pl, G12 8QQ, Glasgow, UK

Author Information

† Authors contributed equally

Corresponding Author

*E-mail: Pablo.docampo@ glasgow.ac.uk

Experimental details:

Most of the experiments were done at Munich University in Germany if not stated otherwise. A small part of the experiments was done at Monash University in Australia.

All the spin-coating procedures were done dynamically if not stated otherwise.

Perovskite synthesis: A 62 wt% perovskite precursor solution with $PbAc_2$ and Methylammonium iodide (MAI) in a 1:3 ratio was prepared in different solvent mixtures and spin-coated for 3 min at 5000 rpm. After spin-coating, the film appears yellow and transparent and turns orange-brown when pre-crystallizes at RT to the perovskite or the precursor phase. After pre-crystallization, the substrate was transferred to the hotplate and annealed at 130 °C for 3 min. To achieve pure orientation in the (200) direction, either a solvent mixture of dimethyl sulfoxide (DMSO) and dimethyl formamide (DMF) was used with 20 vol% DMSO or a combination of THTO/DMF with 13 vol% THTO. To achieve high preference in the (002) direction, a solvent mixture of DMSO/DMF was used with 10 vol% DMSO. Equally strong preference in (200) and (321) directions was achieved with 15 vol% DMSO. No preference in crystal orientation was obtained with pure DMF.

Device fabrication:

Substrate preparation: Fluorine-doped tin oxide (FTO, Pilkington, 7 Ω $\square^{-1}$) substrates were etched with 2 M HCl and zinc powder and cleaned in 2 % Hallmanex detergent, acetone, ethanol and were plasma-cleaned in nitrogen plasma before deposition of charge transport layers. Devices were 2.5cm x 2.5cm giving a total area of 6.25 cm^2 measured using a caliper.

Devices in regular architecture: A sol-gel approach was used to deposit the TiO_2 layer with a solution containing 0.23 M titanium isopropoxide (Sigma-Aldrich, 99.999 %) and 0.013 M hydrochloric acid (HCl) in isopropanol (IPA). The solution was spin-coated dynamically on top of the substrate at 2000 rpm for 45 s, dried at 150 °C for 10 min and annealed at 500 °C for 45 min. Afterwards, the substrates were transferred into a glovebox.

To prepare devices with a C_{60} interface, a monolayer was deposited from a 0.5 mg/mL 4-(1',5'-dihydro-1'methyl-2'H-[5,6]fullereno-C60-Ih-[1,9-c]pyrrol-2'-yl)benzoic acid dissolved in chlorobenzene (CB) with 2000 rpm for 30 s and then annealed at 100 °C for 5 min.

To prepare devices with a [6,6]-phenyl C_{61} butyric acid methyl ester ([60]PCBM, solenne, >99.5%) interface layer on top of TiO_2, the material was dissolved in CB and spin-coated at 2000 rpm for 30 s. A "thin" PCBM layer was achieved with 10 mg/mL concentration and a "thick" PCBM layer with 20 mg/mL concentration.

To achieve a better wetting and nucleation of the perovskite solution, a 0.2 wt% IPA solution of Al_2O_3 nanoparticles (Sigma-Aldrich, < 50 nm particle size, 20wt% in IPA) was deposited on top of PCBM or C_{60} at 2000 rpm for 30 s and dried at 130 °C for 5 min.

The perovskite was deposited on top, and a spiro-OMeTAD (99.6 % purity, Borun New Materials Technology Ltd.) layer afterwards. We used a 75 mg/mL spiro-OMeTAD solution in CB with 3vol% of 170 mg/mL bis(trifluoromethane)sulfonamide lithium salt (Li-TFSI, Sigma-Aldrich) in acetonitrile (Sigma-Aldrich, anhydrous, 99.8%) and 1 vol% 4-*tert*-butyl pyridine (TBP, Sigma-Aldrich, 96%). The solution was spin-coated at 1500 rpm for 45 s. The devices were stored overnight in a desiccator to oxidise the Li-TFSI additive in spiro-OMeTAD. After the overnight storage, the electrode areas on the devices were cleaned with gamma-butyrolactone first and EtOH second and transferred to a GB. To finalise the devices, a 40 nm thick Au counter electrode was thermally evaporated under high vacuum conditions through a metal aperture leading to devices in the range of 0.10 cm^2. The active area was determined with a 0.083 +- 0.001 cm^2 metal aperture.

Devices in regular architecture prepared in Australia

Methylammonium iodide (MAI) was purchased from Greatcell Solar Ltd. 2,2′,7,7′-Tetrakis [N, N-di(4-methoxyphenyl)amino]-9,9′-spirobifluorene (spiro-OMeTAD) was purchased from Luminescence Technology Corp. Glass substrates with a conducting layer of fluorine-doped tin oxide (FTO) of 8 $\Omega\ \square^{-1}$ sheet resistance were purchased from Yingkou Shangneng Photoelectric Material Co., Ltd. All the other materials used in the experiment were purchased from Sigma-Aldrich and used as received.

The FTO glass substrates were pre-patterned by laser and then cleaned with a commercial soap and water, dried with nitrogen, rinsed with ethanol and dried again with nitrogen. Compact-TiO_2 layer and other surface treatments until the perovskite layer deposition was prepared as described above.

For the perovskite layer deposition, a 57 wt% perovskite precursor solution with $PbAc_2$ and Methylammonium iodide (MAI) in a 1:3 ratio was prepared in a DMF/DMSO solvent

mixture. The solvent mixture either contained 10 vol% DMSO for preferred crystal alignment in the (200) direction or 20 vol% DMSO for preferred crystal alignment in the (002) direction, which is reverse to the crystal order achieved with the exact amounts of DMSO described above for the synthesis in Germany. Therefore, the total amount of DMSO has no impact on the resulting perovskite crystal orientation and solar cell device performance but the molar ratio of lead precursor and DMSO in the precursor solution.

The precursor solution was spin-coated for 2 min at 5000 rpm. After spin coating, the film appears yellow/brown and transparent. The substrate was transferred right after spin-coating to the hotplate and annealed at 130 °C for 3 min.

On top of the perovskite layer, we deposited a spiro-OMeTAD layer. We used a 72.5 mg/mL spiro-OMeTAD solution in CB with 1.75 vol% of 170 mg/mL bis(trifluoromethane)sulfonamide lithium salt in acetonitrile and 2.88 vol% 4-tert-butyl pyridine. The solution was spin-coated (static) at 3000 rpm for 45 s. Afterwards, the devices were treated as described above and finalised with an 80 nm Au counter electrode.

Devices in inverted architecture: The poly(3,4-ethylenedioxythiophene): poly(styrenesulfonate) (PEDOT: PSS, Heraeus Clevios, Al 4083) solution was diluted with MeOH in a ratio of 1:2, treated in ultrasonication bath for 10 min and filtered. The solution was spin-coated at 4000 rpm for 45 s and annealed at 150 °C for 10 min. Afterwards, it was immediately transferred to the glove box. The NiOx hole-transporter was deposited via atomic layer deposition (ALD) with a final thickness of 7 nm. To fully oxidise the NiOx layer, we annealed it at 300 °C for 1 h under ambient conditions.

On top of both layers, a 0.2 wt% Al_2O_3 nanoparticle solution in IPA was deposited at 2000 rpm for 30 s and annealed at 130 C for 5 min. The perovskite layer was deposited on top. [6,6]-phenyl C_{61} butyric acid methyl ester ([60]PCBM, solenne, >99.5%) was used for electron transport material with 20 mg/mL solution concentration in CB, which was spin-coated at 1800 rpm for 30 s and annealed at 100 °C for 5 min. A 0.5 mg/mL bathocuproine (BCP, Sigma-Aldrich, 99.99%) solution in IPA, spin-coated at 4000 rpm for 30 s, was used as an interfacial layer between ETM and the electrode. To finalise the devices, a 100 nm thick Ag counter electrode was thermally evaporated under high vacuum conditions through a metal aperture leading to devices in the range of 0.10 cm^2. The active area was determined with a 0.083 +- 0.001 cm^2 metal aperture.

Characterisation:

X-ray diffraction (XRD):

2 ϑ scans were obtained from samples of perovskite deposited on TiO_2-coated FTO glass using an X-ray diffractometer (Bruker D8 Discover in Germany and Bruker D8 Advance in Australia).

Gracing-Incidence-Wide-Angle X-Ray Scattering (GIWAXS) Measurements:

GIWAXS data was acquired with a Ganesha 300XL SAXS-WAXS system with a Cu K_α X-ray source. The incident angle was set to 0.4 °, well above the critical angle of $MAPbI_3$, so the beam penetrated the entire film. The measurement time was ten h if not stated otherwise, and the scattering signal was recorded with a Dectris Pilatus 300k pixel detector with automatic flat-field correction. Data treatment and reduction, including solid-angle, efficiency, polarisation correction and reshaping of 2D images, was conducted with the Matlab software GIXSGUI by Argonne National Laboratory[1]. Like in our previous work[2], further corrections as suggested in the literature were not applied[3,4], which we want to explain in the following: The highly oriented samples presented in the present work gave us the opportunity to check the intensities of (002) and (110) peaks individually. For non-oriented samples, the Debye-Scherrer rings for these planes overlap and are not distinguishable. Here, they could be identified by their slightly differing q values. The intensity for the (110) reflex should be higher than the intensity of the (002) peak. This relation is reversed by applying Lorentz corrections, so simple Lorentz factors for in-plane powders do not apply to these $MAPbI_3$ films[5–7].

Scanning Electron Microscopy (SEM):

A scanning microscope (FEI Helios NanoLab G3 UC) was used to acquire SEM images of perovskite layers prepared on TiO_2-coated FTO glass.

Device Characterization in Germany:

Photovoltaic device performance was measured with a Keithley 2400 source meter in the air at 25 °C under illumination by a Newport Oriel Sol2A solar simulator, calibrated to 100 mW/cm^{-2} with a Fraunhofer ISE certified silicon cell with a mismatch factor of 1.01. The active area of the solar cell was defined with a square metal aperture mask of 0.0831 cm^2.

The *J-V* measurement was obtained in reverse (1.5 to 0V) and forward (0V–1.5 V) scan directions at a scan rate of 200 mV/s with a preconditioning voltage of 1.5V for 10s.

The hysteresis index was calculated according to the following formula from devices in regular architectures:

$$Hysteresis\ index = \frac{PCE(reverse) - PCE(forward)}{PCE\ (reverse)} \qquad (S1)$$

For devices in inverted architectures, the value for *PCE(reverse)* always refers to the higher PCE results during the measurement.

<u>Device Characterization in Australia:</u>

We used a bio logic potentiostat and an Abet Technologies Sun 3000 class AAA with an AM 1.5G spectrum at 100 mW/cm^2 to determine the photovoltaic performance of each solar cell[8]. Non-reflective metal masks with an aperture area of 0.16 cm^2 were used to define the illumination area of the devices. The *J-V* measurement was obtained in reverse (1.2 to −0.1 V) and forward (−0.1–1.2 V) scan directions at a scan rate of 100 mV/s.

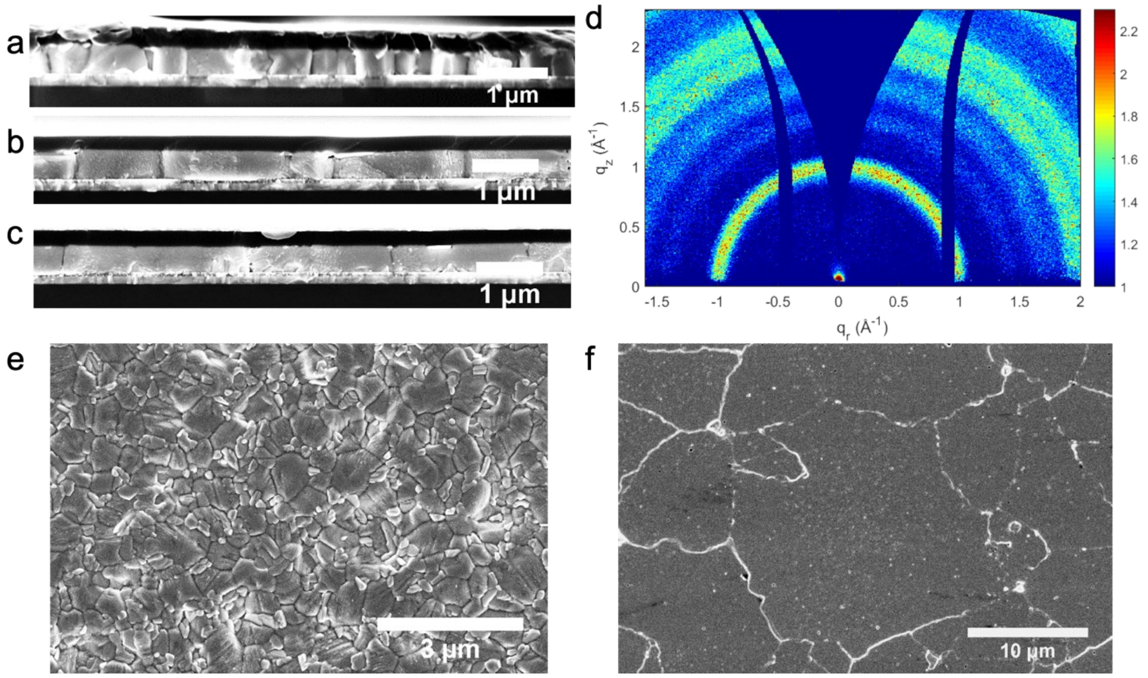

Figure S1: a-c) Cross-sectional SEM images of devices with perovskite films with DMSO in the precursor solution of a) 10 vol%, b) 15 vol% , c) 20 vol%, d) 2D GIWAXS data of film with no preferential orientation synthesized from pure DMF, e) SEM top view of the film from d, f) SEM top view of the (200) facet (THTO) film.

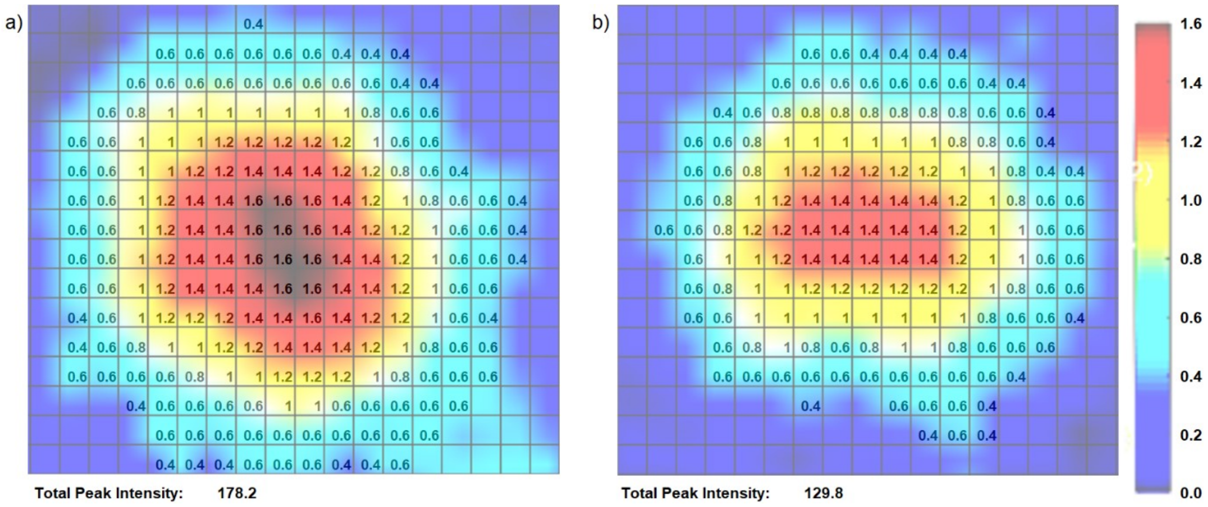

Figure S2: Contrast-enhanced intensity mapping of: a) a (110) peak; and b) a (002) peak; from Fig 1(c) showing their relative intensities. The scale has been adjusted to remove the background signal intensity.

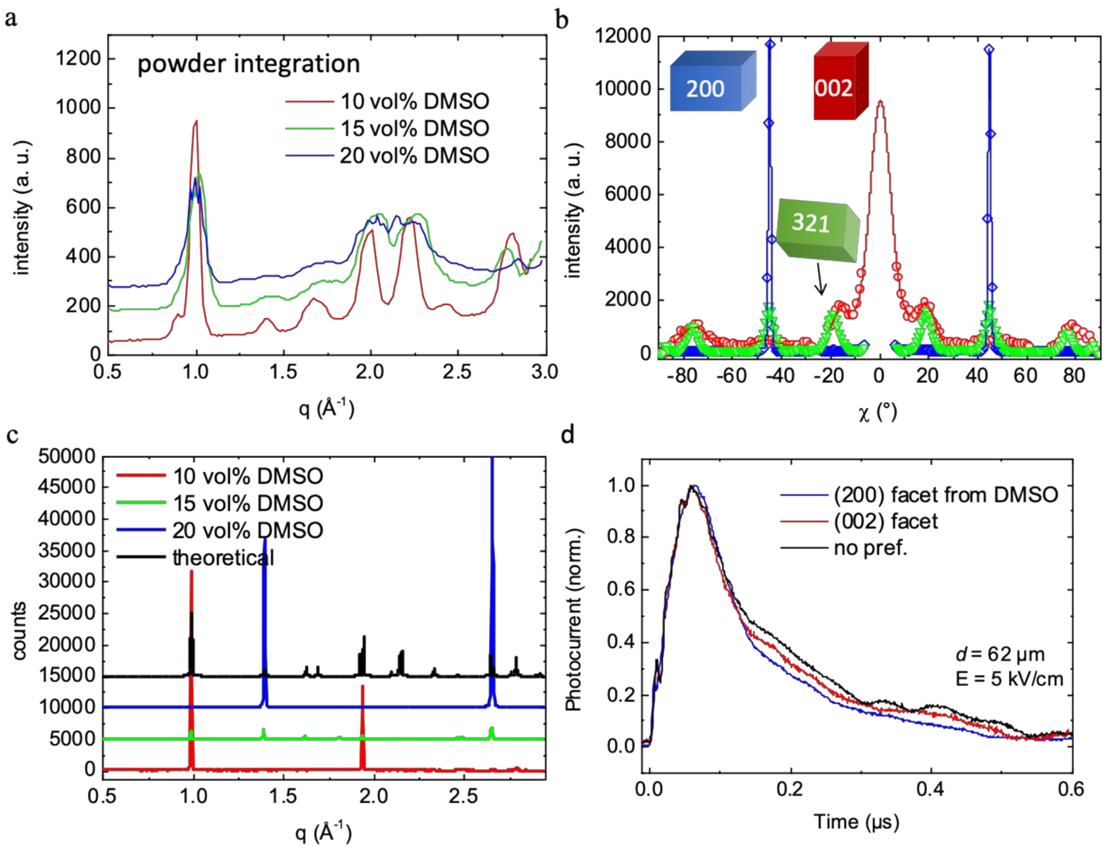

Figure S3: *a) Powder integration from 2D GIWAXS data; b) azimuthal cut at q~1Å^{-1} of 2D GIWAXS data; c) XRD data on thin films investigated with GIWAXS with marked characteristic crystal orientations (200), (321) and (002); d) ToF analysis data of perovskite films prepared from different DMSO concentrations with representative transients at fixed electrode spacing with 62 µm.*

Time of Flight Measurements (ToF):

The generation of charges in the $MAPbI_3$ films was induced upon low-intensity pulsed laser excitation at 540 nm. The laser system consisted of an optical parametric oscillator (OPO), pumped by a solid-state Nd: YAG laser with a repetition rate of 20 Hz and a pulse width of 7 ns. Perovskite thin films, contacted in a lateral architecture and top-coated with a thin PMMA layer to prevent moisture-induced degradation, were illuminated from the semi-transparent glass/gold side at the margin of one contact by focusing the laser through a microscope objective (spot diameter approximately 2 µm). An external DC field was applied through the Au electrodes exclusively during the measurement of the transient (timeframe of about 1 s). The generated photocurrent was monitored with a fast oscilloscope.

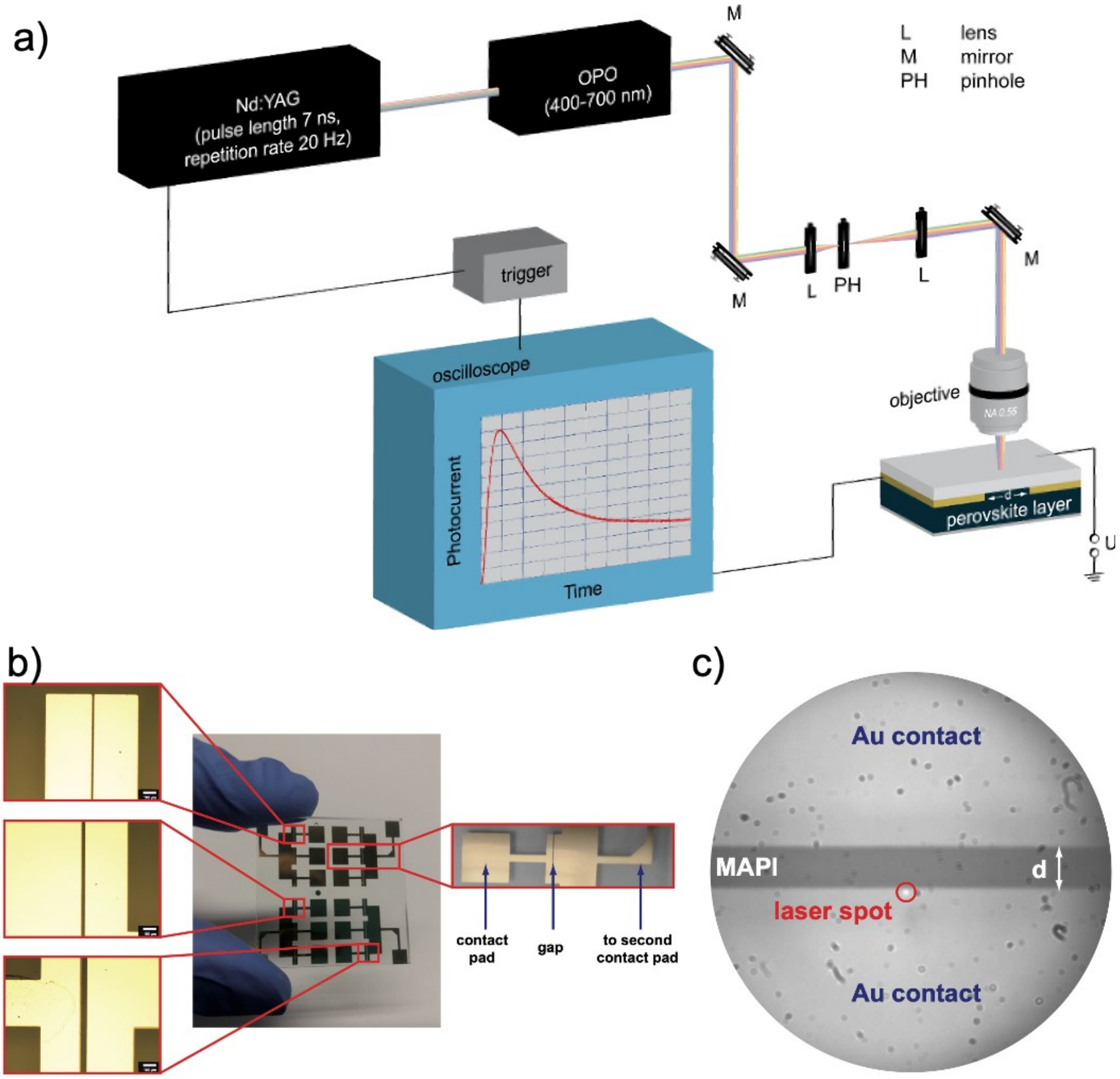

Figure S4: Scheme of the experimental ToF setup; b) photograph of a typical substrate with lateral contact pattern deposited on top and magnified representation as seen through a light microscope; c) top view of a laterally contacted perovskite film through the objective of the ToF setup. The gap filled with perovskite material is clearly visible while the contacts are separated by a defined distance d. Charges are generated in the spot created by the laser light.

TRMC-Measurements:

The perovskite films deposited on quartz or TiO_2 / quartz were mounted in a sealed resonance cavity (8 to 12 GHz) inside an N_2-filled glovebox. The TRMC technique measures the change in microwave power on pulsed excitation (repetition rate 10 Hz). Neutral density filters were used to vary the intensity of the incident light. The normalised laser-induced change in microwave power is related to the change in conductance, ΔG by a sensitivity factor K given by

$$\frac{\Delta P(t)}{P} = -K\Delta G(t) \qquad \text{(S2)}$$

The rise of ΔG is limited by the width of the laser pulse (3.5 ns FWHM) and the response time of the microwave system (18 ns). The slow repetition rate of the laser of 10 Hz ensures full relaxation of all photo-induced charges to the ground state before the next laser pulse hits the sample. Before and during the photoconductance measurements, the samples were not exposed to moisture and air to prevent degradation. By normalising ΔG with the amount of absorbed photons, the product of the yield of free charges, φ and their mobility $\Sigma\mu$ can be derived according to

$$\varphi(\mu_e + \mu_h) = \frac{\Delta G}{F_A I_0 e \beta} \qquad \text{(S3)}$$

Here, I_0 is the number of photons per unit area per pulse, β is the ratio of the inner dimensions of the microwave cell, e is the elementary charge F_A is the fraction of light absorbed by the sample at the excitation wavelength.

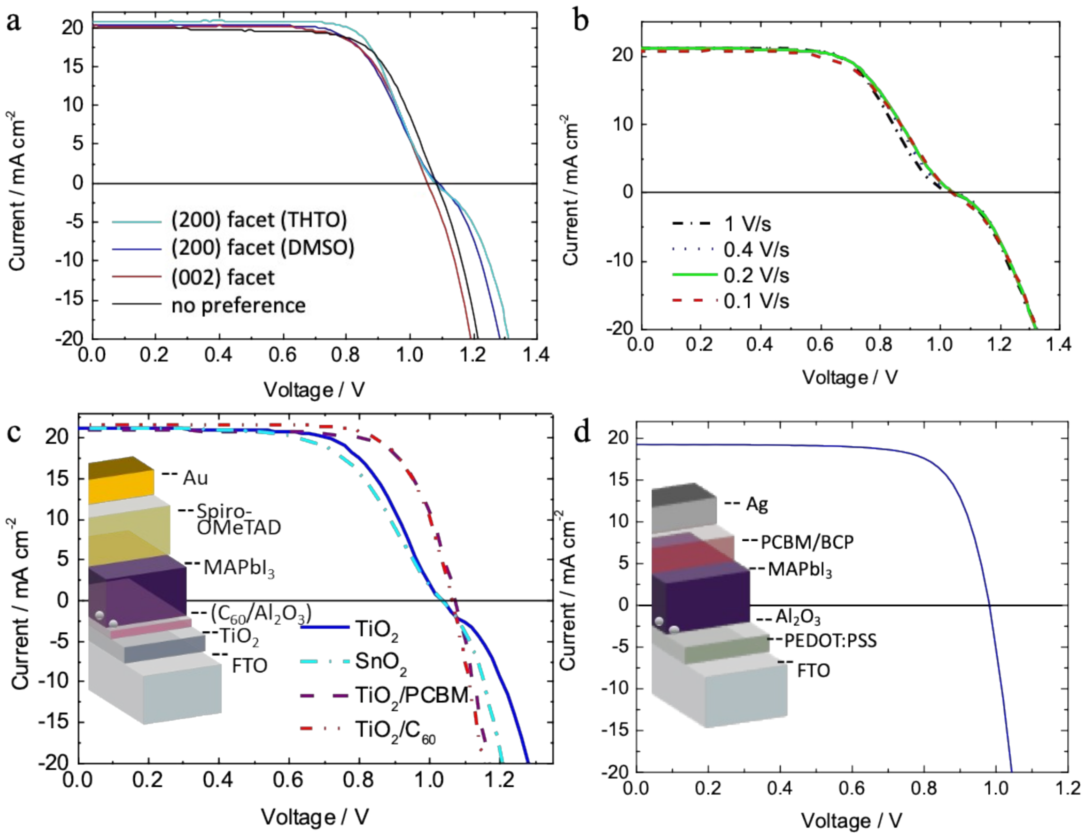

Figure S5: a) J-V curves of FTO/TiO_2/$MAPbI_3$/spiro-OMeTAD/Au devices; b) J-V curves of the (200) facet (THTO) device from a) at varying scan speeds; c) J-V curves of (200) facet (THTO)-based devices with the architecture indicated in the schematics with different ETLs and interfaces; d) inverted devices as indicated in the schematics with (200) facet (THTO) perovskite film.

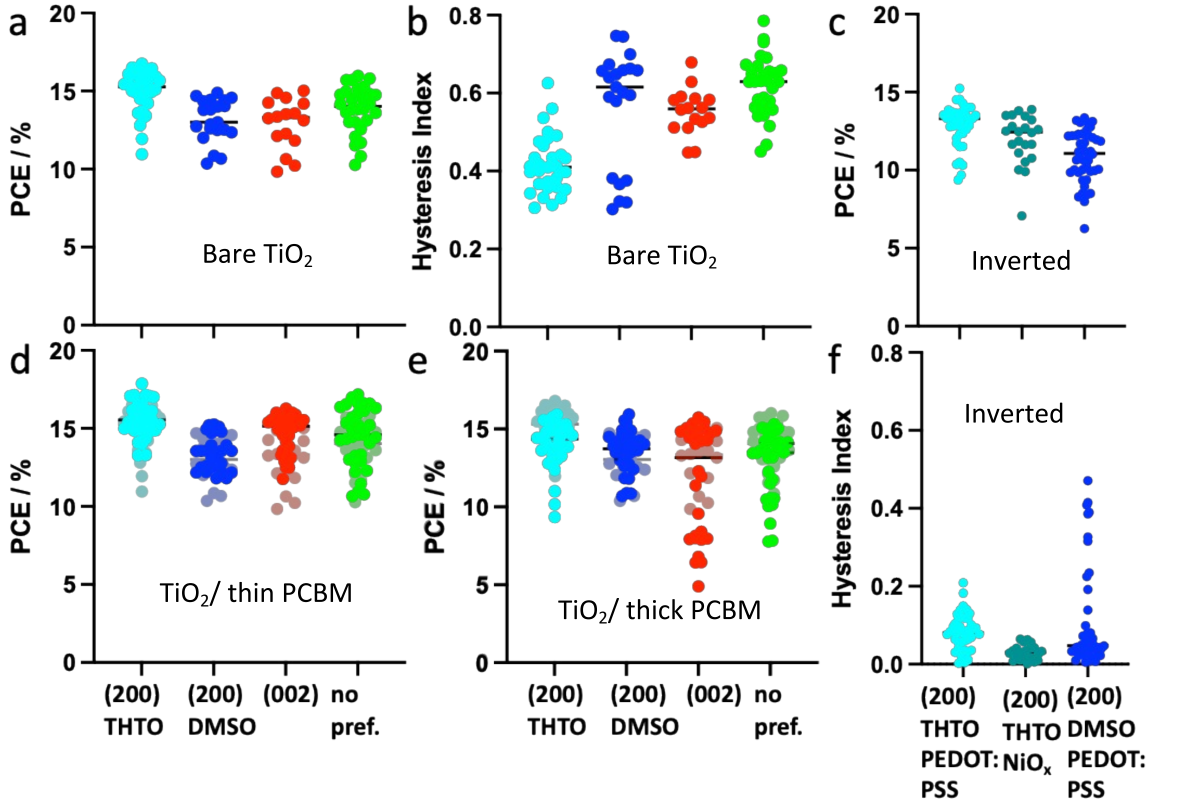

Figure S6: Solar cell performance and hysteresis investigation of devices with differently aligned perovskite grains and in varying device architectures achieved for each value from three different batches; a) PCE values of the reverse scan from devices in regular architectures with TiO_2 bottom interface; b) calculated hysteresis index from devices in regular architectures with TiO_2 bottom interface; c)Solar cell performance from inverted architecture device; d) PCE values of the reverse scan from devices in regular architectures with TiO_2/thin PCBM layer bottom interface and the PCE result from a) in the more saturated colour; e) PCE values of the reverse scan from devices in regular architectures with TiO_2/thick PCBM layer bottom interface and the PCE results from a) in the more saturated colour f) hysteresis investigations of inverted devices with PEDOT:PSS or NiOx as the hole-transport material.

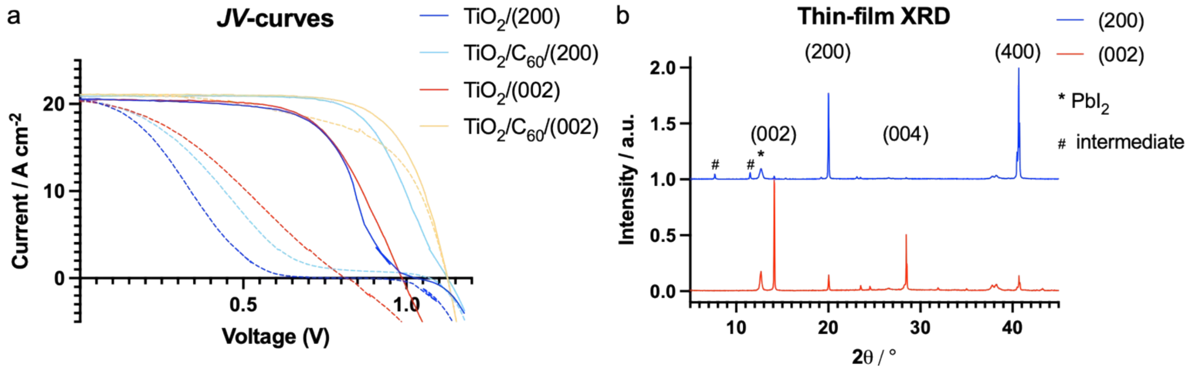

Figure S7: a) J-V-curves FTO/TiO2/MAPbI$_3$/spiro-OMeTAD/Au or FTO/TiO2/C60/MAPbI$_3$/spiro-OMeTAD/Au devices prepared with a 57 wt% perovskite precursor solution and 20 vol% DMSO for (002) or 10 vol% DMSO for (200) preferred facet alignment, straight-line reverse and pointed lines forward scan; b) XRD results of the (200) and (002) facets films from a), the marked intermediate phase agrees with the reported MAI-PbI2-DMSO/DMF phases in [6-8].

Driftfusion Simulations:

Device simulation was performed using a one-dimensional drift-diffusion model named Driftfusion[5]. Devices were first simulated at an equilibrium condition and then a *J-V* simulation was performed. Parameters in the model were chosen to best match experimental conditions. The *J-V* simulation was performed under 0.97 Sun intensity to best match the J_{sc} of the experimental results. An initial forward scan was used to stabilise the device with forward bias before performing a reverse *J-V* scan to best follow the experimental protocol. The solution at the end of the reverse scan was then used as the initial conditions of the forward scan. All *J-V* simulations were performed at a scan speed of 0.2 Vs^{-1}. The key parameters used in the device simulation can be seen in tables S1-S3, and all other parameters were kept the same as the open-source Driftfusion model[5]. It is important to note that the motivation behind the simulations was not to reproduce the exact behaviour of each device but to understand the origin of the s-shapes in the *J-V* curves. To avoid overfitting the experimental data, experimental variables were used as inputs of the model as much as possible; other variables were left as default. To determine the ion mobilities of the different devices, simulations were run with changing ion mobilities. Then, R^2 analysis was performed between the simulated *J-V* scans and the experimental *J-V* scans. Full details of this can be found below.

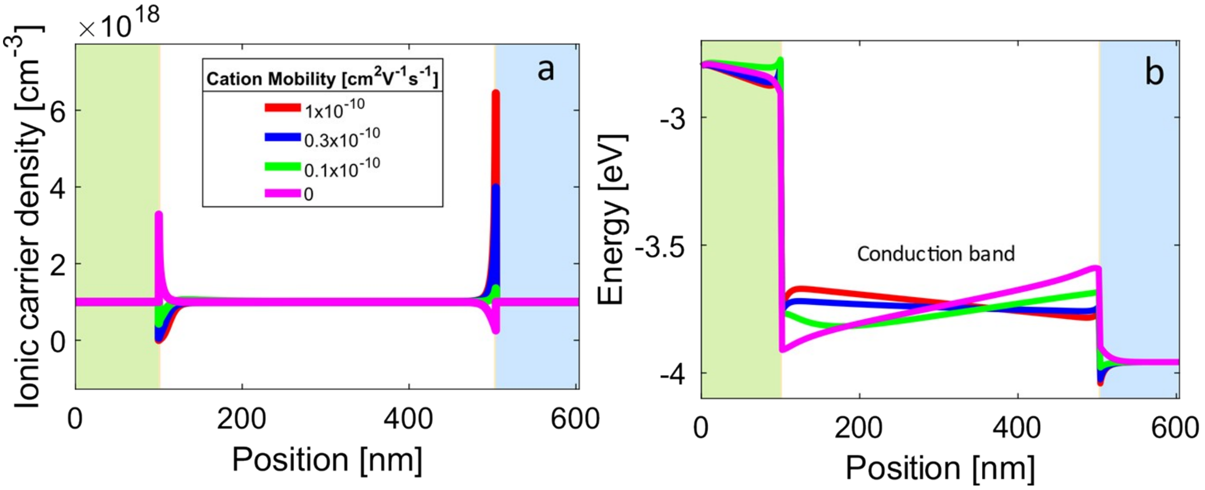

Figure S8: a) Shows the ionic carrier density (cation) as a function of position through a perovskite solar cell device as simulated in Driftfusion at 0.8V during a JV sweep. The mobility at 0 $cm^2V^{-1}s^{-1}$ is a representation of the ionic distribution at a steady state with no applied bias, as the ions do not redistribute during the JV scan. b) shows the conduction band of the solar cell as a function of position with the same variation of cation mobility as shown in Figure S6a.

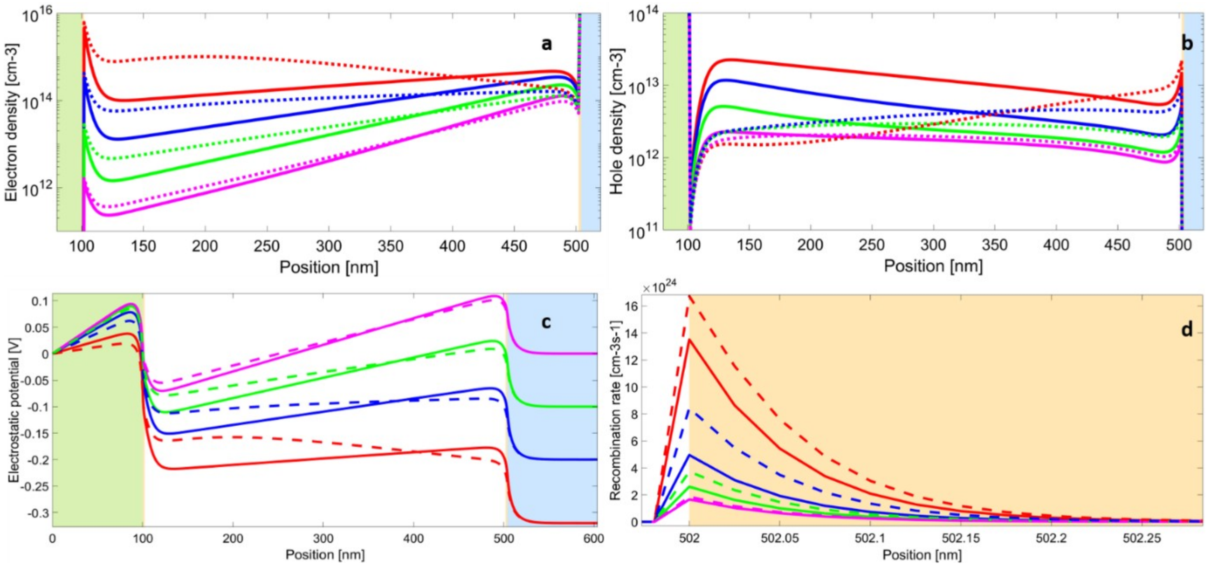

Figure S9: a-d) Solid lines represent 'high' ion mobility (0.96 x10^{-10} $cm^2V^{-1}s^{-1}$) dashed lines represent 'low' ion mobility (0.37x10^{-10} $cm^2V^{-1}s^{-1}$). Red, Blue, Green and Magenta represent 0.6V, 0.7V, 0.8V and 0.9V during the J-V scan, respectively. a) Electron density through the device. b) Hole density through the device. c) Electrostatic potential through the device. d) Recombination rate at the perovskite/TiO^{-2} interface.

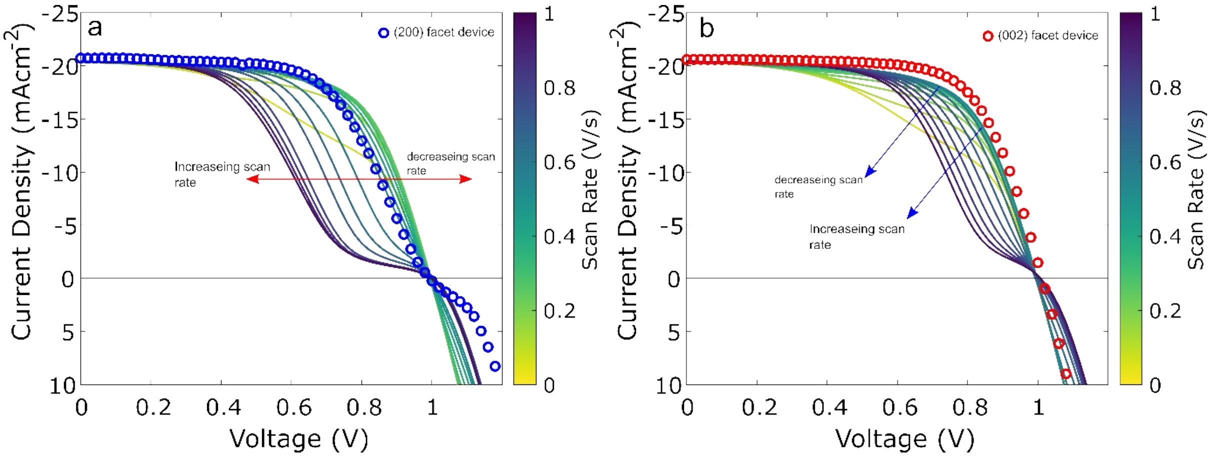

Figure S10: Shows the device results for the (200) and (002) crystal facets in a and b respectively represented as circles. Driftfusion simulations performed with ionic mobilities of 0.37 x 10^{-10} $cm^2V^{-1}s^{-1}$ (figure a) and 0.96 x 10^{-10} $cm^2V^{-1}s^{-1}$ (figure b) with varying scan rates from 0.01V/s to 1 V/s.

<u>R^2 Analysis</u>

To determine the ion mobility to be used in the simulation that would best describe the experimental behaviour, a *coefficient of determination* (R^2) was performed between the simulated results from Driftfusion and the Experimental data. R^2 gives a quantitative result for the agreement between the model and the experimental data

$$R^2 = 1 - \frac{sum\ squared\ regression\ (SSR)}{total\ sum\ of\ squares\ (SST)} = 1 - \frac{\sum (y_i - \hat{y}_i)^2}{\sum y_i - \bar{y})^2} \qquad \text{(S4)}$$

To obtain R^2, MATLABs in the built 'fitlm' function was used to fit the linear regression model and R^2 was determined from this.

The ion mobility with Driftfusion was varied between 0 $cm^2V^{-1}s^{-1}$ and $1.2x10^{-10}$ $cm^2V^{-1}s^{-1}$ with a step size of $0.01x10^{-10}$ $cm^2V^{-1}s^{-1}$. Figure 5 shows the effect of varying the ionic mobility on the *J-V* curve predicted by Driftfusion. At ionic mobilities around and below 0.4 $x10^{-10}cm^2V^{-1}s^{-1}$, there is a pronounced s-shape in the *J-V* curve. At ionic mobilities above this, the *J-V* curve tends to a more 'ideal' shape

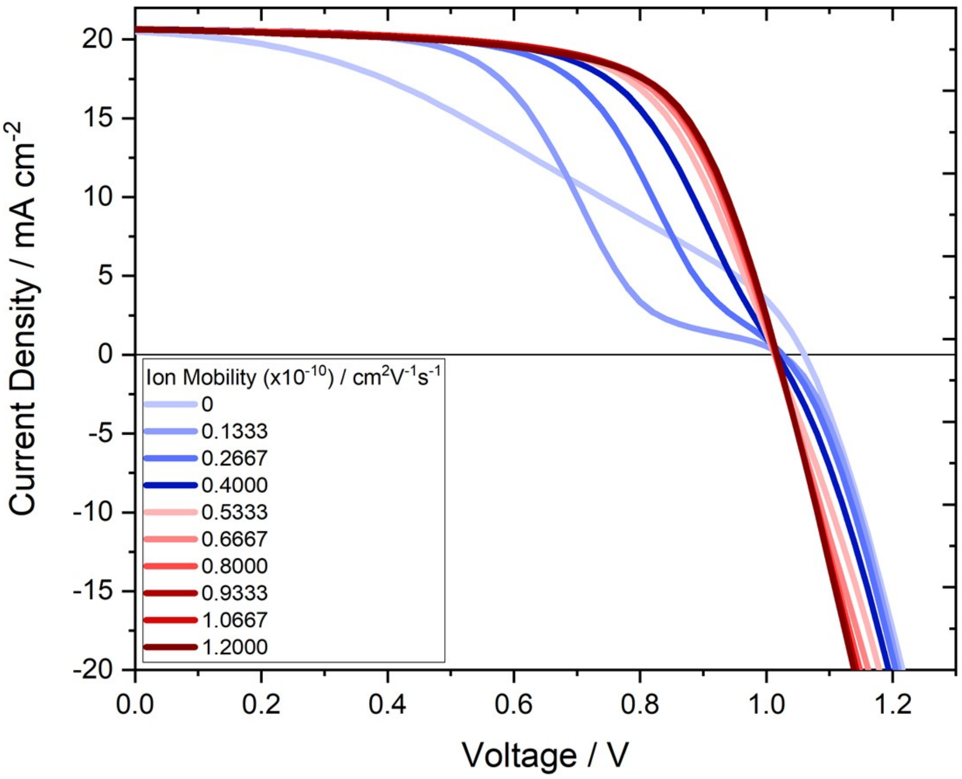

Figure S11: J-V curves for increasing ion mobility between 0$cm^2V^{-1}s^{-1}$ to $1.2x10^{-10}cm^2V^{-1}s^{-1}$

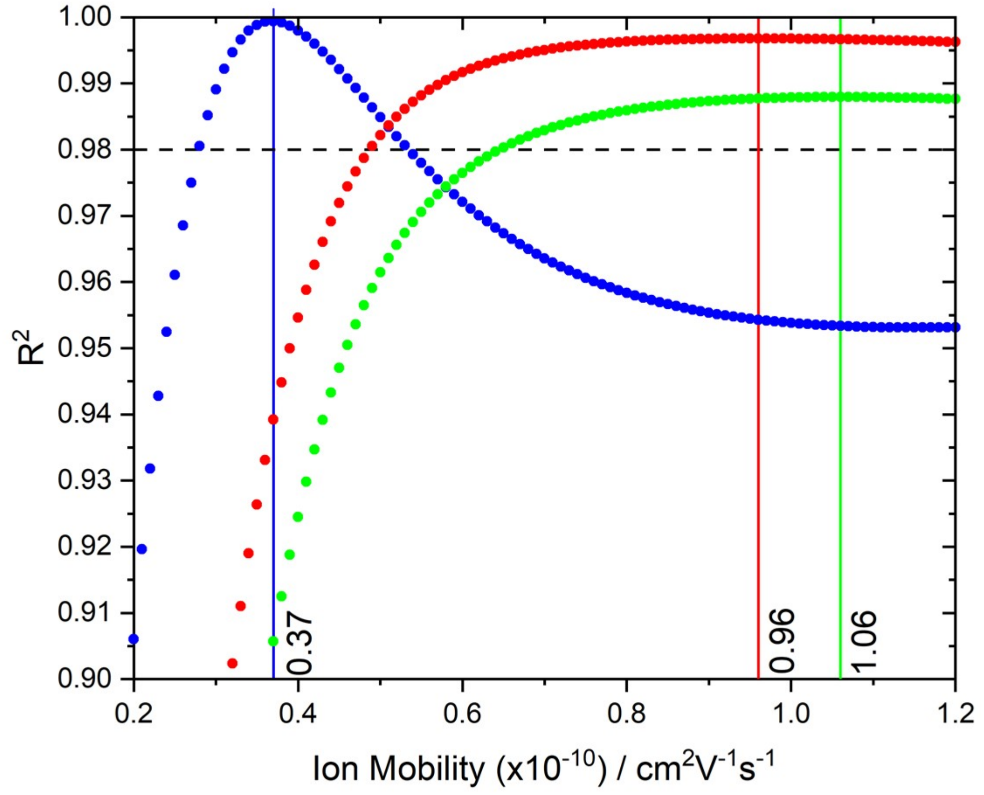

Figure S12: coefficient of determination (R^2) calculated between the simulated J-V curves and the experimental J-V curves as Ion mobility is varied.

This was performed for each of the three different crystal facet orientations over the same ion mobility range. The R^2 value was calculated between the simulated and experimental *J-V* curves above V_{oc} (i.e. when y>0) at each ionic mobility. Figure S8 shows the results of this. Here the axis has been cut to show when the fits are above an R^2 value of 0.9. The blue, red and green dotted curves indicate the R^2 fit for the (002), (200) and mixed (200)(321) facet orientation, respectively. OriginLab's peak fitting tool was used to extract which ionic mobility would give the highest value of R^2. This was determined to be at 0.37 x 10^{-10}, 0.96 x 10^{-10} and 1.06 x 10^{-10} for the (200), (002) and mixed (200)(321) facet orientations respectively. These ion mobilities have an R^2 value of greater than 0.98, as seen by the black horizontal line, and for the (002) and (200) (blue and red), the model accounts for greater than 99% of the variance in the experimental data. It is clear from Figure 6 that there is only a small range of ion mobilities that fit the (002) data, which shows the s-shaped *J-V* curve, agreeing with Figure S7. However, we note that for the (200) and mixed (200)(321) facets, any value above 0.6 x 10^{-10}cm^{2}V^{-1}s^{-1} would reproduce the experimental data with almost the same accuracy.

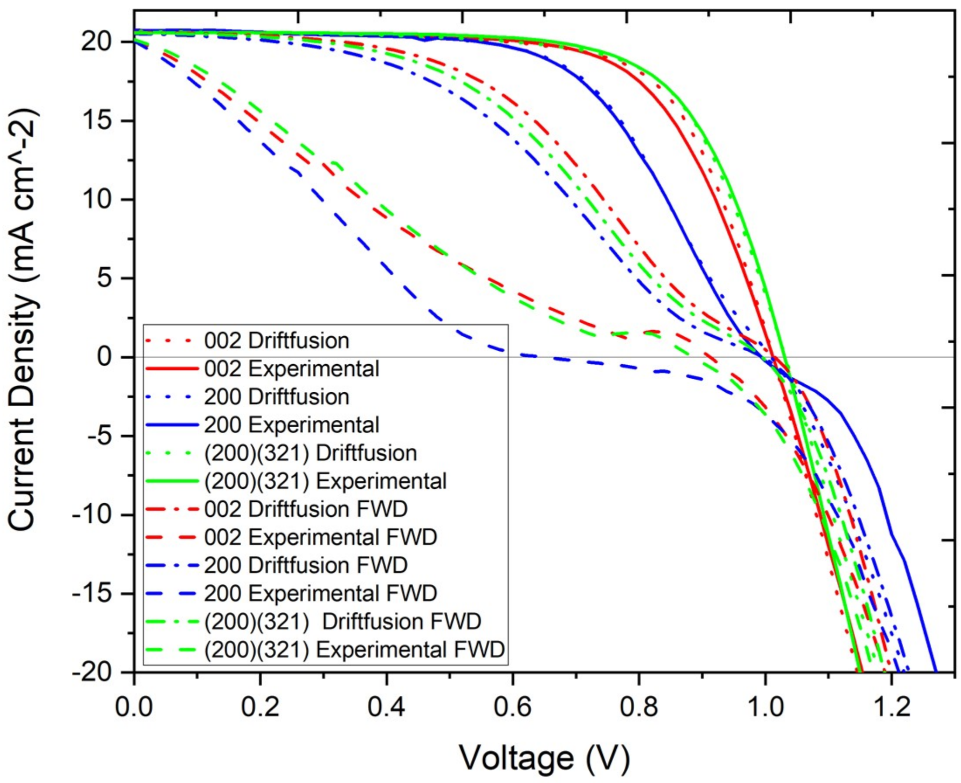

Figure S13: Current Density against voltage for the different orientated facets. Dashed lines show the results from the Diffusion simulation, and the solid lines indicate the experimentally measured results. The dotted line represents the experimentally measured forward J-V scan, and the dash-dot-dash line shows the Driftfusion simulation of the forward scan. The (002) orientated device (red) was simulated with ion mobility of 0.96x10^{-10} $cm^2V^{-1}s^{-1}$. The (200) oriented device (blue) was simulated with ion mobility of 0.37 x10^{-10} $cm^2V^{-1}s^{-1}$. The mixed-orientation device (green) was simulated with ion mobility of 1.06x10^{-10} $cm^2V^{-1}s^{-1}$.

Simulation Parameters

Table S1: Key Parameters in the Perovskite Layer for the different devices

	**Perovskite Layer**		
Key Device Parameters	**(002) facet orientation**	**(200) facet orientation**	**mixed orientation**
Thickness (cm)	4.00E-05	4.00E-05	4.00E-05
Layer points	400	400	400
Electron Affinity (eV)	-3.8	-3.8	-3.8
Ionisation Potential (eV)	-5.4	-5.4	-5.4
Equilibrium Fermi energies (eV)	-4.6	-4.6	-4.6
SRH trap energies (eV)	-4.6	-4.6	-4.6

Density of cation sites cm^{-3}	1.00E+18	1.00E+18	1.00E+18
Density of cation sites cm^{-3}	1.00E+18	1.00E+18	1.00E+18
Electron Mobility ($cm^2V^{-1}s^{-1}$)	1.30E+01	1.30E+01	9.00E+00
Hole Mobility ($cm^2V^{-1}s^{-1}$)	1.20E+01	1.20E+01	8.00E+00
Cation Mobility ($cm^2V^{-1}s^{-1}$)	0.96E-10	0.37E-10	1.06E-10
Anion Mobility ($cm^2V^{-1}s^{-1}$)	0.96E-10	0.37E-10	1.06E-10
Relative Permittivity	2.30E+01	2.30E+01	2.30E+01
Uniform Generation rate ($cm^{-3}s^{-1}$)	2.64E+21	2.64E+21	2.64E+21
Radiative Recombination coefficient	3.60E-12	3.60E-12	3.60E-12

Table S2: Key Parameters in the Spiro-OMeTAD Layer for the different devices

	**Spiro-OMeTAD Layer**		
Key Device Parameters	**(002) facet orientation**	**(200) facet orientation**	**mixed orientation**
Thickness (cm)	1.00E-05	1.00E-05	1.00E-05
Layer points	200	200	200
Electron Affinity (eV)	-2.8	-2.8	-2.8
Ionisation Potential (eV)	-4.9	-4.9	-4.9
Equilibrium Fermi energies (eV)	-4.8	-4.8	-4.8
SRH trap energies (eV)	-4.75	-4.75	-4.75
Density of cation sites cm^{-3}	1.00E+18	1.00E+18	1.00E+18
Density of cation sites cm^{-3}	1.00E+18	1.00E+18	1.00E+18
Electron Mobility ($cm^2V^{-1}s^{-1}$)	5.00E-5	5.00E-5	5.00E-5
Hole Mobility ($cm^2V^{-1}s^{-1}$)	5.00E-5	5.00E-5	5.00E-5
Cation Mobility ($cm^2V^{-1}s^{-1}$)	0.00E+00	0.00E+00	0.00E+00
Anion Mobility ($cm^2V^{-1}s^{-1}$)	0.00E+00	0.00E+00	0.00E+00
Relative Permittivity	4.00E+00	4.00E+00	4.00E+00
Uniform Generation rate ($cm^{-3}s^{-1}$)	0.00E+00	0.00E+00	0.00E+00
Radiative Recombination coefficient	3.18E-11	3.18E-11	3.18E-11

Table S3: Key Parameters in the TiO2 Layer for the different device

	**TiO_2 Layer**		
Key Device Parameters	**(002) facet orientation**	**(200) facet orientation**	**mixed orientation**

Thickness (cm)	1.00E-05	1.00E-05	1.00E-05
Layer points	100	100	100
Electron Affinity (eV)	-4.1	-4.1	-4.1
Ionisation Potential (eV)	-6.4	-6.4	-6.4
Equilibrium Fermi energies (eV)	-4.2	-4.2	-4.2
SRH trap energies (eV)	-4.25	-4.25	-4.25
Density of cation sites cm^{-3}	1.00E+18	1.00E+18	1.00E+18
Density of cation sites cm^{-3}	1.00E+18	1.00E+18	1.00E+18
Electron Mobility ($cm^2V^{-1}s^{-1}$)	1.00E-01	1.00E-01	1.00E-01
Hole Mobility ($cm^2V^{-1}s^{-1}$)	1.00E-01	1.00E-01	1.00E-01
Cation Mobility ($cm^2V^{-1}s^{-1}$)	0.00E+00	0.00E+00	0.00E+00
Anion Mobility ($cm^2V^{-1}s^{-1}$)	0.00E+00	0.00E+00	0.00E+00
Relative Permittivity	1.20E+01	1.20E+01	1.20E+01
Uniform Generation rate ($cm^{-3}s^{-1}$)	0.00E+00	0.00E+00	0.00E+00
Radiative Recombination coefficient	1.54E-10	1.54E-10	1.54E-10

References

1. Jiang, Z. *GIXSGUI* : a MATLAB toolbox for grazing-incidence X-ray scattering data visualization and reduction, and indexing of buried three-dimensional periodic nanostructured films. *J Appl Crystallogr* **48**, 917–926 (2015).
2. Oesinghaus, L. *et al.* Toward Tailored Film Morphologies: The Origin of Crystal Orientation in Hybrid Perovskite Thin Films. *Adv Mater Interfaces* **3**, 1600403 (2016).
3. Baker, J. L. *et al.* Quantification of Thin Film Crystallographic Orientation Using X-ray Diffraction with an Area Detector. *Langmuir* **26**, 9146–9151 (2010).
4. DeLongchamp, D. M., Kline, R. J. & Herzing, A. Nanoscale structure measurements for polymer-fullerene photovoltaics. *Energy Environ Sci* **5**, 5980 (2012).
5. Cao, J. *et al.* Identifying the Molecular Structures of Intermediates for Optimizing the Fabrication of High-Quality Perovskite Films. *J Am Chem Soc* **138**, 9919–9926 (2016).
6. Gratia, P. *et al.* The Many Faces of Mixed Ion Perovskites: Unraveling and Understanding the Crystallization Process. *ACS Energy Lett* **2**, 2686–2693 (2017).
7. Tan, W. L. & McNeill, C. R. X-ray diffraction of photovoltaic perovskites: Principles and applications. *Appl Phys Rev* **9**, 021310 (2022).
8. Surmiak, M. A. *et al.* High-Throughput Characterization of Perovskite Solar Cells for Rapid Combinatorial Screening. *Solar RRL* **4**, 2000097 (2020).
